# Elevated Gut Microbiome-Derived Propionate Levels Are Associated With Reduced Sterile Lung Inflammation and Bacterial Immunity in Mice

**DOI:** 10.3389/fmicb.2019.00159

**Published:** 2019-02-14

**Authors:** Xiaoli Tian, Judith Hellman, Alexander R. Horswill, Heidi A. Crosby, Kevin P. Francis, Arun Prakash

**Affiliations:** ^1^Department of Anesthesia and Perioperative Care, University of California, San Francisco, San Francisco, CA, United States; ^2^Department of Immunology and Microbiology, Anschutz Medical Campus, University of Colorado, Aurora, CO, United States; ^3^Preclinical Imaging, PerkinElmer, Hopkinton, MA, United States; ^4^San Francisco General Hospital, University of California, San Francisco, San Francisco, CA, United States

**Keywords:** lung injury, short-chain fatty acids, SCFA, acetate, propionate, IR, inflammation

## Abstract

Short-chain fatty acids (SCFA) are important dietary and microbiome metabolites that can have roles in gut immunity as well as further afield. We previously observed that gut microbiome alteration via antibiotics led to attenuated lung inflammatory responses. The rationale for this study was to identify gut microbiome factors that regulate lung immune homeostasis. We first investigated key factors within mouse colonic lumen filtrates (CLF) which could elicit direct inflammatory effects *in vitro*. We identified lipopolysaccharide (LPS) and SCFAs as key CLF ingredients whose levels and inflammatory capacity changed after antibiotic exposure in mice. Specifically, the SCFA propionate appeared to be a key regulator of LPS responses *in vitro*. Elevated propionate: acetate ratios, as seen in CLF after antibiotic exposure, strongly blunted inflammatory responses *in vitro*. *In vivo*, exposure of lungs to high dose propionate, to mimic how prior antibiotic exposure changed SCFA levels, resulted in diminished immune containment of *Staphylococcus aureus* pneumonia. Finally, we discovered an enrichment of propionate-producing gut bacteria in mice with reduced lung inflammation following lung ischemia reperfusion injury *in vivo*. Overall, our data show that propionate levels can distinctly modulate lung immune responses *in vitro* and *in vivo* and that gut microbiome increased production of propionate is associated with reduced lung inflammation.

## Introduction

The human body coexists with a vast commensal microbiome that is increasingly recognized to play important roles in human health, physiology, and disease (Cho and Blaser, 2012; Shreiner et al., 2015; Sender et al., 2016; Alverdy and Krezalek, 2017; Young, 2017). Interactions between the microbiome and host are best understood at specific interfaces, such as the gut, oral cavity and the skin. Additionally, there appears to be a prominent role for gastrointestinal dysbiosis and pathobionts in the etiology of inflammatory bowel disease, other intestinal disorders, and critical illness in general (Alverdy and Krezalek, 2017). From an evolutionary viewpoint, human physiology has evolved closely with specific gut microbiota which, prior to the advent of antibiotics, were likely not exposed to sudden perturbations in composition over the life cycle of the human host (after early post-natal colonization). These symbiotic organisms may serve purposes besides food digestion and vitamin production, such as during immune education and host defense. While it may be conceptually challenging at first to explain how gut commensal microbial communities affect physiological processes in distant organ systems, a few studies have supported this paradigm (Trompette et al., 2014; Vieira et al., 2015, 2016).

We and others have reported that the gut microbiome influences lung inflammatory responses and these data support the current hypothesis for the existence for a gut-lung axis of communication (Trompette et al., 2014; Marsland et al., 2015; Prakash et al., 2015; Samuelson et al., 2015; Budden et al., 2017). However, the key questions of how communication along this axis occurs still remain unanswered. We speculate that commensal-associated molecular patterns (CAMPs) and metabolites in concert modulate lung inflammatory responses by either directly acting on resident lung immune cells or indirectly through immune reprogramming of circulating immune cells or both. Released CAMPs and metabolites may transit through the intestinal mucosa and portal circulation and travel to the lungs via the liver and heart. Since the pulmonary and gastrointestinal systems are both exposed to environmental and infectious threats, it is plausible that the commensal microbial community influences these two systems. This concept is supported by our earlier published data that lung alveolar macrophages from mice with antibiotic-altered gut microbiota were less responsive to inflammatory ligands than their unaltered counterparts (Prakash et al., 2015). The gut-lung axis may contribute to establishing lung immune homeostasis that could be akin to a baseline state of immune tonicity or injury readiness (reviewed in Lloyd and Marsland, 2017). By understanding the impact of commensal-derived factors on lung immunity, it may be possible to selectively or temporarily regulate lung immunity: for example, by bolstering host defenses during pneumonia or mitigating sterile pneumonitis after gastric acid aspiration.

In this study we focused on identifying factors produced by the gut microbiome that could regulate lung responses to sterile and infectious injuries. We discovered that antibiotic-mediated dysbiosis led to large alterations in SCFA levels, specifically, propionate and acetate levels. Furthermore, propionate appeared to have a concentration-dependent ability to modify LPS inflammatory responses *in vitro*, with low and high concentrations augmenting and blocking inflammatory responses, respectively. High propionate:acetate ratios appeared to skew LPS responses toward less inflammation while low ratios appeared to skew LPS responses toward more inflammation *in vitro*. This was confirmed by the *in vivo* observation that direct administration of high propionate concentrations to mouse lungs resulted in worsening of an experimental pneumonia, likely through the inhibition of beneficial inflammation needed to fight the infection. Finally, examining the microbiomes of mice with varying degrees of sterile lung injury revealed that high propionate-producing bacteria (*Lachnospiraceae*) were enriched significantly in mice that exhibited low lung inflammation phenotypes.

Overall, our study strongly suggests that gut commensal bacteria continuously prime resident cells in distant organ systems, such as the lungs, through shed toxins and specific metabolite compositions. Furthermore, alterations in gut microbiome composition can profoundly change inflammatory responses to sterile injury and pathogenic infectious responses. We had previously shown that reducing lung inflammation results in a deficient host immune response to infection (Tian et al., 2017). Being able to control and modulate lung inflammatory responses through gut microbiome or metabolite manipulation could aid in preventing pulmonary complications from prolonged hospitalization and antibiotic exposure in critically ill patients.

## Materials and Methods

### Animals

Male mice (12–15 weeks old) were either purchased (The Jackson Laboratory, Bar Harbor, ME, United States) or bred at the animal facility at University of California, San Francisco. Purchased mice were allowed to acclimatize to their new housing for at least 1 week before any experiments on them were conducted. Wild-type C57BL/6 and C3H/HeOuJ mice were used in this study.

Only male mice were used in our experiments primarily to reflect the fact that trauma disproportionately affects human males. Based on our previous studies, we used group sizes of 6–10 for all experiments (Prakash et al., 2012, 2015). All mice for a given experiment were either littermates or purchased/bred such that they were age-matched. Mice used in these experiments were randomly chosen either to undergo the various surgeries (sham vs. IR) or treatments (+/- specific treatments); therefore there was no attempt made to blind the individuals conducting the experiments. However, in situations where mice received a treatment or control before IR surgery or infection after surgery, the individual collecting the organs/plasma and generating the ELISA data was unaware of which mice received which specific treatment.

### Reagents and Cell Lines

Short-chain fatty acids (SCFA) (acetate, butyrate, propionate, formate), Trichostatin A, polymyxin B, lipopolysaccharide (LPS), and lipoteichoic acid (LTA) were purchased from Sigma-Aldrich, St. Louis, MO, United States. Pam3CysSKKK and FSL-1 were both obtained from EMC Microcollections, Tubingen, Germany. The following cell lines were used in this study: HUVEC (primary human umbilical vein endothelial cells, used at passage 6 or less, Promocell, Heidelberg, Germany), EOMA (129 background endothelial cell line, ATCC, Manassas, VA, United States), SVEC-40 (C3H/HeJ TLR4 mutant background endothelial cell line, ATCC), and MH-S (wild-type BALB/c alveolar macrophage cell line, ATCC).

### Antibiotic Treatment

A group of wild-type C3H/HeOuJ background mice were treated with antibiotic containing water *ad libitum* or control group was given standard drinking water. Antibiotic treatment consisted of neomycin and polymyxin B (final concentrations: 0.6 mg/mL for neomycin and 120 units/mL for polymyxin B, both from Sigma-Aldrich, St. Louis, MO, United States) given in drinking water for 8 weeks as described earlier (Prakash et al., 2015). This combination of antibiotics was chosen specifically for the ability of both to remain within the gut and not be absorbed into circulation; by using this combination to target the gut microbiome, we could focus primarily on its effects without disruption of other microbiomal niches. Supplementary Figure S6 demonstrates the effects of this combination on the richness, evenness, and diversity of the microbiome after 7 weeks of treatment.

### Colonic Lumen Filtrate (CLF)

Stool from mice was obtained in two ways: by scruffing mice and collecting stool as it was produced by the mice; or by collecting small and large intestines of mice and expressing it out of the intestinal cavity. Homogenized stool was then sterile filtered to create CLF and protein concentration measured by bicinchoninic acid (BCA) method. Protein concentrations of CLF were measured using standard assay (Pierce^TM^ BCA Protein Assay Kit, Thermo Fisher Scientific, Waltham, MA, United States). CLF sterility was confirmed by plating on LB agar plates. Stool from cages of mice (3 cages, 15 mice total per group) that received either control or antibiotic water were collected, pooled, and CLF was prepared. CLF was added to tissue culture supernatant for various experiments described in the concentrations noted.

### SCFA and Amino Acid Measurement

Stool or colonic lumen filtrate (prepared as described above) was sent to PennCHOP metabolomics core facility^1^ for SCFA and amino acid analysis. CLF was further filtered using 1.2, 0.65, and 0.22 μm filter plates (Millipore, Billerica, MA, United States). The filtrate was loaded into total recovery vials (Waters, Milford, MA, United States) for analysis. Short chain fatty acids were quantified using a Water Acquity uPLC System with a Photodiode Array Detector and an autosampler (192 sample capacity). Samples were analyzed on a HSS T3 1.8 μm 2.1 × 150 mm column. The flow rate was 0.25 mL/min, the injection volume was 5 μL, the column temperature was 4°C, the sample temperature was 4°C, and the run-time was 25 min per sample. Eluent A was 100 mM sodium phosphate monobasic, pH 2.5, eluent B was methanol, the weak needle wash was 0.1% formic acid in water, the strong needle wash was 0.1% formic acid in acetonitrile, and the seal wash was 10% acetonitrile in water. The gradient was 100% eluent A for 5 min, gradient to 70% eluent B from 5 to 22 min, and then 100% eluent A for 3 min. The photodiode array was set to read absorbance at 215 nm with 4.8 nm resolution. Samples were quantified against standard curves of at least five points run in triplicate. Standard curves were run at the beginning and end of each metabolomics run. Quality control checks (blanks and standards) were run every eight samples. Results were rejected if the standards deviate by greater than ± 5%. Concentrations in the samples were calculated as the measured concentration minus the concentration of the solvent; the range of detection was at least 1 – 100 μmol/g stool.

### Ventilated Lung Ischemia Reperfusion (Unilateral Left Pulmonary Artery Occlusion) Surgery

A mouse model of unilateral left pulmonary artery (PA) occlusion was used, as we have described previously (Prakash et al., 2012). Briefly, anesthetized mice (using IP tribromoethanol (Avertin^®^); Sigma-Aldrich) were orally intubated, given buprenorphine (IP; Harry Schein, Melville, NY, United States), and placed on a mini-vent mouse ventilator (Harvard Apparatus, Holliston, MA, United States), using tidal volumes of 0.225 mL (7.5 mL/kg), and a respiratory rate of 180 breaths/min (assuming an average mouse weight of 30 g). A left thoracotomy via the interspace between the 2nd and 3rd ribs was performed and the left PA was identified and ligated using a slip knot suture with 7-0 or 8-0 prolene monofilament suture. The end of the suture was externalized through a narrow bore (27 g) needle to the anterior chest wall. Prior to closure of the thorax, the left lung was reinflated with positive end expiratory pressure (PEEP). Local anesthetic (3-4 drops of 0.25% bupivacaine) was applied topically prior to skin closure. The total period of mechanical ventilation and surgery was approximately 20–25 min. After skin closure, mice were extubated and allowed to recover from anesthesia. After 60 min of ischemia, the ligature on the PA was released and left lung reperfusion started. At the experimental end-point times, mice were euthanized and the blood and lungs were collected.

Blood was collected from anesthetized mice via cardiac puncture using a heparinized syringe, centrifuged (14,000 *g*, 5 min) and the plasma separated, flash frozen in liquid nitrogen and stored at -80°C. Lower portions of the left lungs were excised and placed in Trizol^®^ (Thermo Fisher Scientific, Waltham, MA, United States) at -80°C for RNA isolation. Levels of cytokines and chemokines (described later) were quantified in plasma.

Mice received equivalent durations of mechanical ventilation (20–25 min), and were left spontaneously breathing during their recovery from anesthesia and the remainder of the ischemia period and subsequent reperfusion or equivalent periods in the sham mice.

While this lung IR procedure has high initial survival rates of 80–90% on average, some mice die from irreparable damage to the PA or left bronchus during the slip-knot placement. Mice that did not survive the surgery or the reperfusion period due to technical complications in the surgical procedure (predominantly, left bronchus or left PA injury) were excluded from the study. The overall attrition rate was 10–20%.

### Lung Injury Scoring

Lung injury was scored in histology images by one of two methods: semiquantitative visual scoring and by ImageJ analysis of the images for counts of inflammatory cells and %area occupied. The former (semi-quantitative) scoring method (1 = no lung injury and 5 = severe lung injury) was performed as previously described (Prakash et al., 2015). The latter (quantitative ImageJ) scoring method was performed follows: in brief, the ImageJ freehand selection tool was used to trace the perimeter of each region of interest. The area extending beyond the perimeter of the vessel was cleared, and the color threshold of the image was adjusted using the default method with the following parameters: hue = 0–255, saturation = 0–255, brightness = 130–255, threshold color = white, background = dark, color space = HSB. The image was converted to an 8-bit gray scale, and the threshold was adjusted using the B&W defaults and a range of 0–150. Counts were outlined and summarized using the analyze particles window (size = 0-infinity, circularity = 0.0–1.00). Average percent Area was also calculated. Lung Injury Scores and cutoffs for high vs. low lung injury designation for this study are included in Supplementary Figure S7.

### *S. aureus* Experimental Pneumonia

C57BL/6 wild-type mice were pretreated with high (1 mM) or low (0.1 mM) propionate intratracheally (IT) 2 h prior to IT administration (10^8^ CFU) of luminescent strain of *S. aureus* (Newman-lux strain generously provided by Alex Horswill, University of Colorado, Denver). IT administration was done under isoflurane anesthesia and with direct visualization. Six hours after infection, mice were live imaged using IVIS^®^ *in vivo* imaging system (see below for more details). Lungs were then collected and imaged *ex vivo* using IVIS^®^ and luminescence was also measured using Cytation5 cell imaging multi-mode reader (BioTek, Winooski, VT, United States).

*Staphylococcus aureus* (Newman-lux strain) was grown as follows: after an overnight inoculation in LB broth, serial dilutions of the overnight stock was grown at 37C in a 24-well tissue culture dish with orbital shaking in the Cytation5 cell imaging multi-mode reader (BioTek, Winooski, VT, United States). Every 5 min, an OD reading and a luminescence reading (to verify healthy growth of the luminescence producing strain) were obtained. When the mice were ready for infection, the wells that were at OD 0.3 (mid-log phase) were removed and used for IT infection. Later CFU measurements of these innocula provided the actual CFU count administered to the mice as described above.

### *In vivo* Imaging (IVIS^®^)

C57BL/6 mice after IT propionate and *S. aureus* administration were imaged at the time points noted. Imaging was conducted on the IVIS^®^ Spectrum Instrument (PerkinElmer, Hopkinton, MA, United States) as previously described (Zhang et al., 2013). Luminescence imaging was performed with an open filter for 5 min.

### Sandwich Enzyme-Linked Immunosorbant Assay (ELISA)

Levels of IL-6 produced were determined using the corresponding mouse duoset or Quantikine kits (R&D Systems, Minneapolis, MN, United States). A multiplex ELISA to measure an immune panel of cytokines was used to identify the pattern of expression of other inflammatory and associated cytokines. This measurement of protein levels of cytokines and chemokines were performed once with a 20plex immune array kit (Thermo Fisher Scientific, Waltham, MA, United States). Analytes included in the panel: FGFβ, IL-1β, IL-10, IL-13, IL-6, IL12, IL-17, MIP-1α, GMCSF, MCP-1, IL-5, VEGF, IL-1α, IFNγ, TNFα, IL-2, IP-10 (CXCL10), MIG, KC, IL-4. Those analytes not shown in Supplementary Figure S2 were detected at low levels or below the level of detection of the assay. All assays were performed according the manufacturer’s supplied protocol. All ELISA measurements (except for the multiplex immune panel) were repeated 2–3 times from independently conducted experiments and representative data shown. Standard curves were generated and used to determine the concentrations of individual cytokines or chemokines in the sample.

### Microbiome Analysis

Single stool pellets from 23 wild type C3H/HeOuJ mice: 11 that received (Neo/PMB) or 12 that received control water for 8 weeks were processed by the UCSF Colitis and Crohn’s Disease Microbiome Research Core Facility as previously described (Fujimura et al., 2016; Mar et al., 2016). Briefly, 46 mouse fecal samples (23 from the week prior to starting antibiotic/control water and 23 from the week prior to lung IR surgery) were processed for DNA extraction, PCR amplification of the V4 hypervariable region of the 16S rRNA gene, and DNA sequencing on the Illumina NextSeq. DNA was extracted from all samples using a modified CTAB extraction protocol. Each DNA sample was PCR amplified in triplicate using primers that (1) targeted the V4 hypervariable region of the 16S rRNA gene, (2) contained a unique barcode sequence to enable demultiplexing of pooled samples, and (3) contained an adapter sequence that enables the amplicon to bind to the NextSeq flow cell. Successful amplicons were pooled in equimolar concentrations and sequenced on the Illumina NextSeq.

Downstream analysis: Merged sequencing read pairs containing less than two expected errors were binned into OTUs (operational taxonomic units) using a 97% sequence similarity threshold. OTUs determined to be chimeric or not of bacterial origin were discarded. Additionally, OTUs known to be common contaminants observed in greater than 50% of extraction controls were discarded and the maximum read count of each remaining OTU in any single extraction control was subtracted from the reads counts of that OTU for all sample. Read counts for OTUs which summed across all samples that were less than 1/1000th of a percent of the total read count for the entire dataset were discarded to minimize noise. Sample read numbers were representatively rarefied to 17,444 reads resulting in a rarefied OTU table. Forty-five of 46 (98%) samples had quality filtered read numbers above the specified rarefying threshold and were included in the downstream analyses.

#### DNA Extraction

Individual murine fecal samples were placed into lysing matrix E (LME) tubes pre-aliquoted with 500 of hexadecyltrimethylammonium bromide (CTAB) DNA extraction buffer and incubated at 65°C for 15 min. An equal volume of phenol:chloroform:isoamyl alcohol (25:24:1) was added to each tube and samples were homogenized in a Fast Prep-24 homogenizer at 5.5 m/s for 30 s. Tubes were centrifuged for 5 min at 16,000 × *g* and the aqueous phase was transferred to individual wells of a deep-well 96-well plate. An additional 500 μl of CTAB buffer was added to the LME tubes, the previous steps were repeated, and the aqueous phases were combined. An equal volume of chloroform was mixed with each sample, followed by centrifugation at 3000 × *g* for 10 min to remove excess phenol. The aqueous phase (600 μl) was transferred to a deep-well 96-well plate, combined with 2 volume-equivalents of polyethylene glycol (PEG) and stored overnight at 4°C to precipitate DNA. Plates were centrifuged for 60 min at 3000 × *g* to pellet DNA and the PEG solution was removed. DNA pellets were washed twice with 300 μl of 70% ethanol, air-dried for 10 min and suspended in 100 μl of sterile water. DNA samples were quantitated using the Qubit dsDNA HS Assay Kit and diluted to 10 ng/μl.

#### DNA Amplification and Sequencing

The V4 region of the 16S rRNA gene was amplified in triplicate as previously described (see citation below). Triplicate reactions were combined and purified using the SequalPrep Normalization Plate Kit (Invitrogen) according to manufacturer’s specifications. Purified amplicons were quantitated using the Qubit dsDNA HS Assay Kit and pooled at equimolar concentrations. The amplicon library was concentrated using the Agencourt AMPure XP system (Beckman-Coulter) quantitated using the KAPA Library Quantification Kit (KAPA Biosystems) and diluted to 2 nM. Equimolar PhiX was added at 40% final volume to the amplicon library and sequenced on the Illumina NextSeq 500 Platform on a 153 bp × 153 bp sequencing run.

#### OTU Table Generation

Raw sequence data was converted from bcl to fastq format using bcl2fastq v2.16.0.10. Paired sequencing reads with a minimum overlap of 25 bp were merged using FLASH v1.2.11. Index sequences were extracted from successfully merged reads and demultiplexed in the absence of quality filtering in QIIME (Quantitative Insights Into Microbial Ecology, v1.9.1), and reads with more than two expected errors were removed using USEARCH’s fastq filter (v7.0.1001). Remaining reads were de-replicated at 100% identity, clustered into operational taxonomic units (OTUs) at 97% sequence identity, filtered to remove chimeric sequences, and mapped back to OTUs using USEARCH v8.0.1623. Taxonomy was assigned using the Greengenes database (May 2013). OTUs detected in Negative Extraction Controls (NECs) were considered potential contaminants and filtered as follows: any known common contaminant OTU present in more than half of the NECs for this study was removed from all samples; the maximum read count for any OTU found in fewer than half of the NECs was subtracted from all samples; and any remaining OTU with a total read count less than 0.001% of the total read count across all samples was removed.

#### Alpha-Diversity

Alpha-diversity indices were computed in QIIME. Comparisons between mouse genotypes were assessed using the Kruskal–Wallis one-way analysis of variance test. Results with a *p*-value of < 0.05 were considered statistically significant.

#### Beta-Diversity

Beta-diversity dissimilarity matrices (Bray-Curtis, Canberra, weighted and unweighted uniFrac distances) were generated in QIIME. Variables were assessed for their relationship to bacterial beta-diversity by permutational analysis of variance (PERMANOVA) using the Adonis function (vegan package) in the R environment; variables of *p* < 0.05 were considered statistically significant.

#### Taxonomic Differences

Enriched taxa were identified using a “three model” approach where Poisson, negative-binomial, and zero-inflated negative-binomial models were applied to each taxon individually, and the model that minimized the Akaike information criterion value (AIC) was selected for each taxon. Before applying the models, the OTU table was de-noised by removing taxa that were present in fewer than 25% of the samples. To adjust for multiple-testing, the false-discovery rate was calculated for each taxon; a *q*-value of < 0.20 was considered significant.

### Statistical Analysis

Data in the figures are expressed as mean ±SD. Data from *in vivo* studies comparing two conditions were analyzed using two-tailed unpaired non-parametric Mann–Whitney analyses. Data from *in vitro* studies comparing two conditions were analyzed using two-tailed unpaired parametric t test with Welch’s correction. GraphPad Prism was used for statistical analyses (GraphPad Software, La Jolla, CA, United States). For all *in vivo* and *in vitro* experiments, *p* values < 0.05 were considered statistically significant. *P-*values are represented as follows in the figures: ^∗^ < 0.05; ^∗∗^ < 0.01; ^∗∗∗^ < 0.001; ^∗∗∗∗^ < 0.0001. For multiple comparisons, one-way ANOVA was used, with *p*-values represented as follows: α < 0.05; αα < 0.01; ααα < 0.001; αααα < 0.0001. When comparing treatment conditions against an untreated or control condition (indicated in figure legends), two-tailed unpaired parametric *t*-test with Welch’s correction was used, with *p*-values represented as follows: δ < 0.05; δδ < 0.01; δδδ < 0.001; δδδδ < 0.0001. Experiments were repeated two or more times, as indicated in the figure legends.

### Study Approval

All mouse studies were approved from an ethical and methodological standpoint by the Institutional Animal Care and Use Committee (IACUC) at the University of California, San Francisco and followed the ARRIVE guidelines.

## Results

### Colonic Lumen Filtrate (CLF) Is Pro-inflammatory and Contains Lipopolysaccharide (LPS)

**FIGURE 1:**
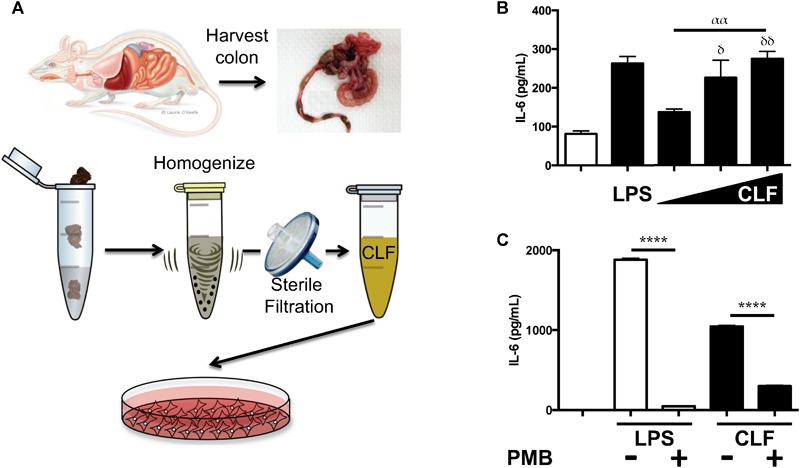
Colonic Lumen Filtrate stimulates (CLF) cells similarly to LPS and contains LPS. **(A)** Schematic for preparation of CLF from mouse stool. **(B)** Human endothelial cells (EC) were stimulated with LPS or CLF (increasing doses from 1 to 100 mg/mL) and IL-6 measured. **(C)** LPS or CLF stimulation of mouse alveolar macrophages (AM) were both inhibited with polymyxin B (PMB) treatment. *P-*values are represented as follows: ^∗^ < 0.05; ^∗∗^ < 0.01; ^∗∗∗^ < 0.001; ^∗∗∗∗^ < 0.0001. For multiple comparisons using one-way ANOVA, *p*-values are represented as follows: α < 0.05; αα < 0.01; ααα < 0.001; αααα < 0.0001. For comparisons against an untreated or control condition, *p*-values are represented as follows: δ < 0.05; δδ < 0.01; δδδ < 0.001; δδδδ < 0.0001. All experiments were conducted at least twice and representative data are shown.

After previously reporting that antibiotic treatment led to diminished inflammatory changes in the mouse lung following lung ischemia reperfusion (Prakash et al., 2015), we tested whether colon contents of mice contained inflammation-inducing factors. CLF from wild-type C57BL/6 mice was prepared (Figure 1A) and used to stimulate human endothelial cells (HUVEC). Similar to LPS, CLF challenge resulted in dose-dependent IL-6 production (Figure 1B). Interestingly, CLFs pro-inflammatory effects on human cells were poorly blocked by polymyxin B (PMB – binds and inactivates LPS) (Supplementary Figure S1). However, in murine alveolar macrophages (AMs), PMB was able to block the majority of CLF inflammatory effects (Figure 1C). Additionally, like LPS, CLF effects were also blocked both in HUVEC and AMs by histone deacetylase (HDAC) inhibition both by high dose SCFA (butyrate) as well as trichostatin A (TSA) (Supplementary Figure S2). Taken together, CLF likely contains both LPS as well as other inflammatory agents produced or shed by the gut commensal microbiome.

### CLF From Antibiotic-Treated Mice Is Less Inflammatory and Contains Less LPS

**FIGURE 2:**
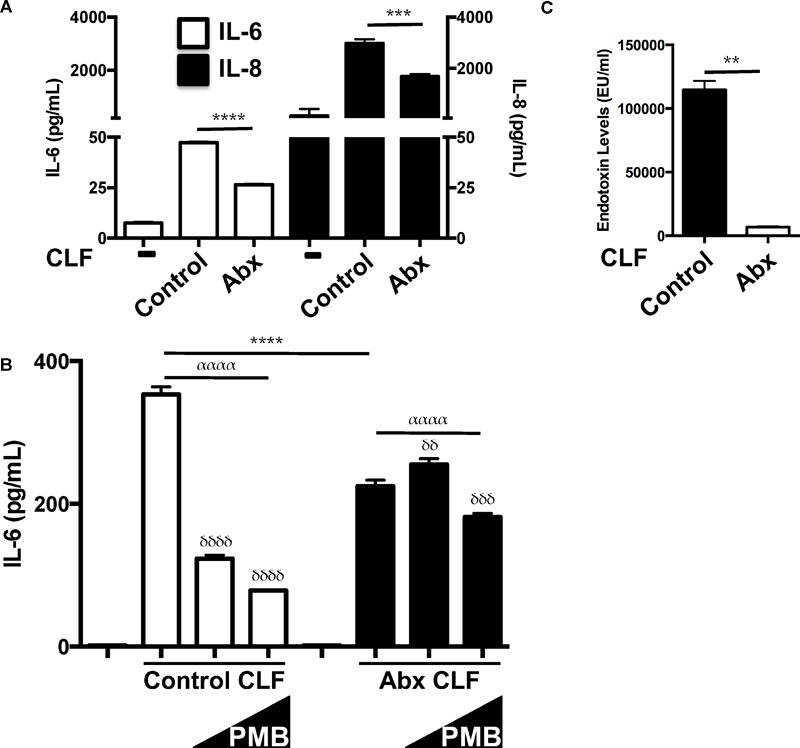
CLF from antibiotic-treated mice is less inflammatory and contains less LPS. **(A)** HUVEC (EC) respond less to antibiotic-exposed CLF (50 mcg/mL) vs. control CLF (50 mcg/mL) vis-à-vis IL-6 and IL-8 production. **(B)** Mouse alveolar macrophages (AM) treated with antibiotic-exposed CLF (100 mcg/ml) respond differently to polymyxin B (PMB; 10 ng/mL and 10 mcg/mL) inhibition compared to control CLF (100 mcg/mL) vis-à-vis IL-6 production. **(C)** Endotoxin levels in control vs. antibiotic-treated mouse stool measured by LAL assay. *P-*values are represented as follows: ^∗^ < 0.05; ^∗∗^ < 0.01; ^∗∗∗^ < 0.001; ^∗∗∗∗^ < 0.0001. For multiple comparisons using one-way ANOVA, *p*-values are represented as follows: α < 0.05; αα < 0.01; ααα < 0.001; αααα < 0.0001. For comparisons against an untreated or control condition [e.g., in **(B)** CLF treatment without any polymyxin B], *p*-values are represented as follows: δ < 0.05; δδ < 0.01; δδδ < 0.001; δδδδ < 0.0001. All experiments were conducted at least twice and representative data are shown.

Since antibiotic-treated mice displayed blunted inflammatory responses to sterile lung injury and diminished alveolar macrophage responses to LPS (Prakash et al., 2015), we investigated the effects of CLF from these mice. CLF from antibiotic-treated mice (2-week treatment) generated less inflammation from both HUVEC (Figure 2A) and murine AMs (Figure 2B). Consistently, antibiotic CLF contained greatly reduced levels of LPS (Figure 2C). Furthermore, while control CLF was blocked by PMB, antibiotic CLF was not (Figure 2B). Overall, these data suggest that antibiotic treatment altered the composition of CLF and reduced its stimulatory effect partially due to a significant reduction in LPS levels. This is consistent with the fact that the antibiotics chosen have primarily excellent gram negative coverage.

### Short-Chain Fatty Acid (SCFA) and Amino Acid Metabolomic Analysis of CLF Reveals Antibiotic-Induced Changes in Acetate and Propionate Levels

**FIGURE 3:**
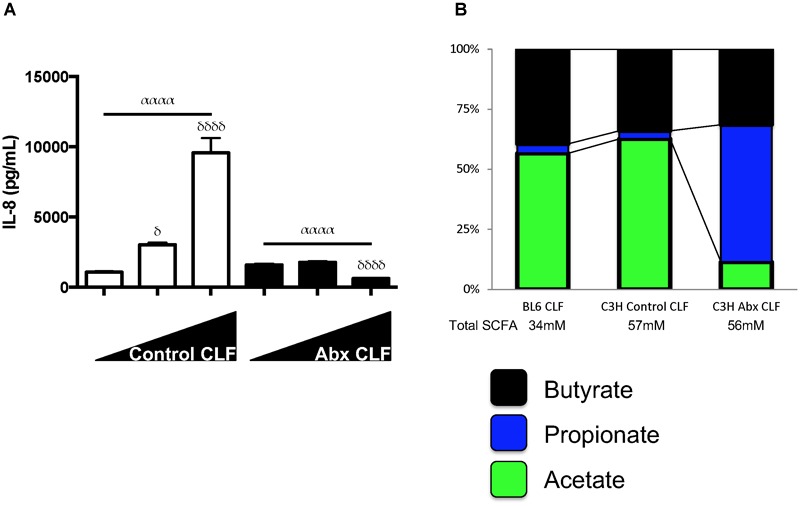
SCFA levels can effect highly divergent inflammatory responses and CLF from control mice and antibiotic-treated mice have different propionate:acetate ratios. **(A)** Increasing dosage of CLF from control or antibiotic-treated animal stool stimulates or inhibits HUVEC inflammatory cytokine (IL-8) production. HUVECs (EC) were treated with increasing amounts of control or antibiotic-exposed CLF (10 mcg/mL, 50 mcg/mL, and 100 mcg/mL) and IL-8 levels measured by ELISA. **(B)** SCFA levels were measured in WT C57BL/6 and WT C3H/HeOuJ mouse stool collected from cages of cohoused mice (5 mice for WT C57BL/6 and 15 mice for control and antibiotic-treated C3H/HeOuJ mice) receiving either control or antibiotic water and pooled before CLF was prepared. Figure shows only on acetate, propionate and butyrate levels. For multiple comparisons using one-way ANOVA, *p*-values are represented as follows: α < 0.05; αα < 0.01; ααα < 0.001; αααα < 0.0001. For comparisons against an untreated or control condition (e.g., 10 mcg/mL CLF treatment), *p*-values are represented as follows: δ < 0.05; δδ < 0.01; δδδ < 0.001; δδδδ < 0.0001.

While comparing the effects of LPS and CLF, we noted the similarities in responses to HDAC inhibition by millimolar levels of the SCFA, butyrate, as described earlier. However, we also noted that lower dose (micromolar) butyrate displayed a paradoxical effect in enhancing LPS mediated and CLF-mediated inflammation for IL-6 and other specific inflammatory genes (Supplementary Figure S3). This paradoxical effect was more pronounced during stimulation with lower doses of LPS (data not shown). Furthermore, when we challenged HUVEC to increasing levels of antibiotic CLF, it appeared that antibiotic CLF contained inhibitory factors in contrast to control CLF (Figure 3A). Since SCFAs could alter the inflammatory potential of LPS, we hypothesized that perhaps high levels of an inhibitory SCFA were present in antibiotic CLF that could reconcile with the corresponding diminished lung inflammation we had previously observed *in vivo* (Prakash et al., 2015). CLF itself contains SCFAs as well as other metabolites, so we measured SCFA and amino acid levels present in CLF samples used thus far (Wild-type C57BL/6 CLF, Control C3H/HeOuJ CLF, Antibiotic C3H/HeOuJ CLF) (Supplementary Table ST1). Focusing on three important SCFAs, namely, acetate, propionate and butyrate, we noted that antibiotic treatment did not change butyrate levels, but instead drastically altered the propionate:acetate ratio within CLF (Figure 3B). Similarly to butyrate, propionate can also act as an HDAC inhibitor at millimolar concentrations (Chang et al., 2014).

### Propionate Effects on LPS-Exposed Cells *in vitro* Also Depend on Concentration and Requires LPS Sensing by TLR4

**FIGURE 4:**
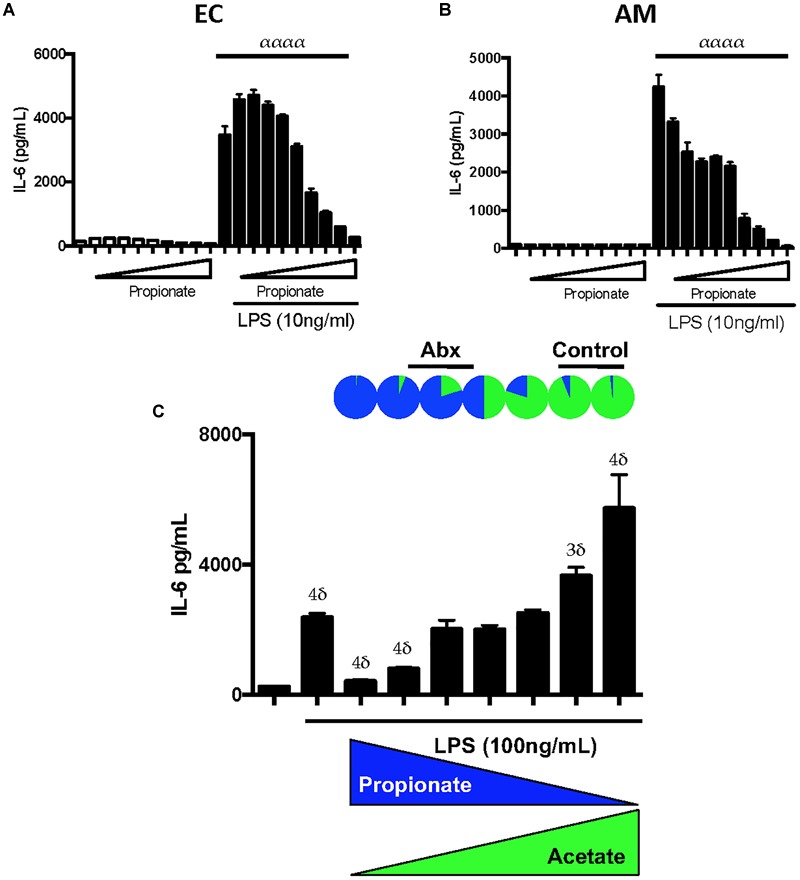
Propionate can have paradoxical effects on LPS inflammatory responses similar to butyrate and propionate:acetate ratios found in control and antibiotic-exposed CLF augment and inhibit LPS-mediated inflammation, respectively. EOMA Endothelial cells (EC) **(A)**, and MH-S Alveolar Macrophage (AM) **(B)** cell lines were treated with a wide dose range of propionate (25 μM–6.4 mM) either in the presence or absence of LPS (10 ng/mL) and overnight production of IL-6 by ELISA was measured. **(C)** AMs were challenged with LPS and inverse levels propionate:acetate, ranging from 4 0mM:10 μM (rightmost) to 10 μM:40 mM (leftmost); therefore, from left to right, propionate was 40, 20, 10, 5, 2.5, 1.25, 0.625, 0.3125 mM, 160, 80, 40, 20, 10 μM, and acetate vice versa. The relative proportions propionate:acetate in control CLF is represented at top as a pie chart and approximate ranges of ratios that correspond to antibiotic CLF and control CLF are noted: Control CLF ratios of propionate:acetate (1:19) as well as estimated ratios in peripheral circulation (1:99; see Supplementary Figure S7) and antibiotic CLF ratios of propionate:acetate (5:1) as well as estimated ratios in peripheral circulation (5:4; see Supplementary Figure S7). IL-6 levels were measured in the supernatant by ELISA after overnight treatment. For multiple comparisons using one-way ANOVA **(A,B)**, *p*-values are represented as follows: α < 0.05; αα < 0.01; ααα < 0.001; αααα < 0.0001. For comparisons against an untreated or control condition (e.g., LPS only treatment, **C**), *p*-values are represented as follows: δ < 0.05; 2δ < 0.01; 3δ < 0.001; 4δ < 0.0001. All experiments were conducted at least twice and representative data are shown.

We next tested the effects of low- and high-dose propionate on LPS responses in mouse AMs and ECs. We observed that low and high-dose propionate effects on ECs and AM LPS responses largely mirrored the effects of low and high-dose butyrate (Figure 4A,B), namely, low-dose propionate was able to augment LPS inflammatory responses and high-dose blunted LPS responses. This effect was dependent on TLR4 and not caspase-11 sensing of LPS (Supplementary Figure S4). TLR2 ligands’ inflammatory effects could also be similarly modulated by low dose and high dose SCFAs (Supplementary Figure S5). When we used caspase-11 and TLR4 mutant ECs to study CLF inflammatory effects in the presence of low and high-dose SCFAs, we noted that SCFA effects were largely dependent on the ability of cells to recognize extracellular LPS through TLR4 (data not shown). This indicated to us that LPS (and/or other gut microbiome-derived TLR4 ligands) is likely the major bacterial ligand present in our CLFs.

### Propionate:Acetate Ratios Can Result in Augmentation or Reduction of LPS Inflammatory Responses *in vitro*

SCFAs are not uniformly diluted into the systemic circulation after absorption in the gut. In fact, various groups have estimated the levels absorbed by the transit organs between the colonic contents and systemic circulation (Cummings et al., 1979, 1987; Boets et al., 2015). Based on the estimates for intestinal and liver absorption reported by Boets et al. (2015), namely 95% butyrate absorption and 90% propionate, respectively, we estimated the propionate:acetate ratio in the peripheral circulation for control and antibiotic-treated mice (Supplementary Figure S6). To examine the inflammation modulating effects of these SCFAs in circulation and in the lung, we exposed mouse lung macrophages *in vitro* to LPS in the presence of varying propionate:acetate ratios. We hypothesized that low propionate:acetate ratios would augment the LPS responses while high ratios would inhibit inflammation. After subjecting AMs to LPS in conjunction with inverse ratios of propionate and acetate, we observed exactly that (Figure 4C). Therefore, these *in vitro* data strikingly recapitulated our *in vivo* observations (Prakash et al., 2015) that antibiotic-treatment in mice that targeted mostly gram-negative bacteria within the gut microbiome and resulted in a switch from low to high propionate:acetate ratios within CLF, which in turn, similarly altered lung inflammatory responses *in vivo* and *in vitro*.

### *In vivo* Lung Pretreatment With High Dose Propionate Results in Worsening of *S. aureus* Pneumonia

**FIGURE 5:**
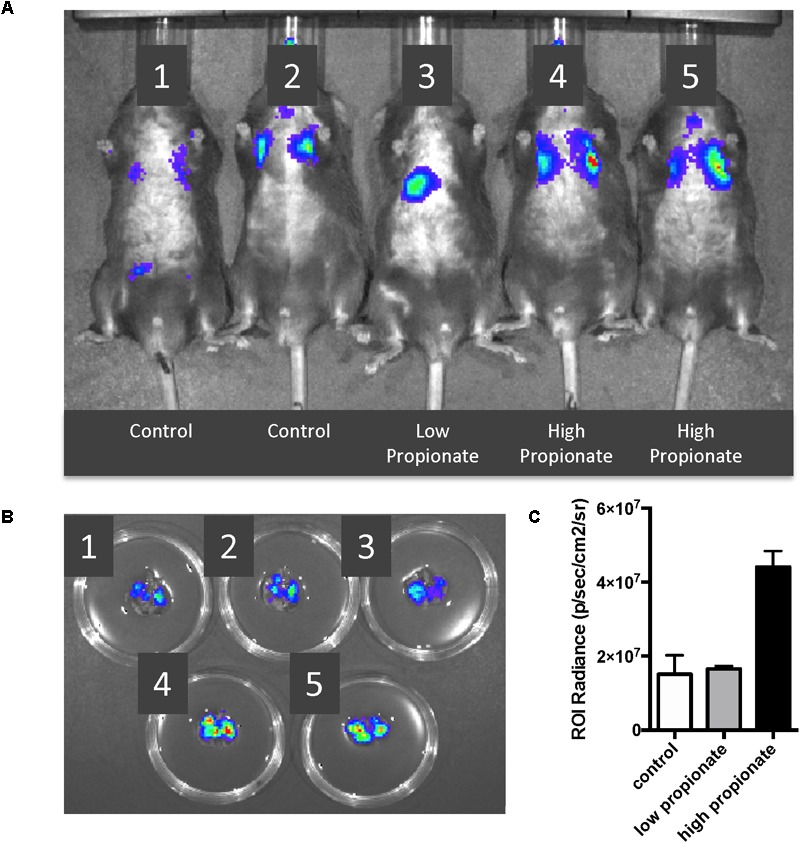
High dose propionate pre-treatment negatively impacts control of *S. aureus* pneumonia. C57BL/6 wild-type mice were pre-treated with high (1 mM) or low (0.1 mM) dose propionate for 2 h, following which they were infected with 10^8^ CFU of luminescent *S. aureus*. **(A)** IVIS^®^ *in vivo* imaging of control (Shreiner et al., 2015; Sender et al., 2016), low dose (Alverdy and Krezalek, 2017), and high dose (Cho and Blaser, 2012; Young, 2017) propionate pre-treated mice, 6 h after *S. aureus* infection. **(B)** Immediately after imaging in **(A)**, mice were euthanized and lungs harvested and imaged using IVIS^®^. **(C)** Levels of luminescence quantified by measurement of luminescence [Region of interest (ROI) radiance]. This experiment as performed twice and combined data are shown in **(C)**.

Acetate levels within the peripheral circulation likely remain constant given their use and production by most gut microbiome species and the lack of major absorption by the intestine and liver (in contrast to propionate and butyrate). Therefore to establish *in vivo* significance to our *in vitro* propionate findings, we chose to address the question of whether altering propionate levels in the lung could affect lung immune responses. We pretreated C57BL/6 wild type mice with high and low dose propionate [1 and 0.1 mM, intratracheally (IT)] and 2 h later subjected them to a *S. aureus* pneumonia with 10^8^ CFU (IT). We had previously examined the kinetics of this infection and found that maximal infection was detected 6 h after inoculation and at 24 h the infection had resolved (data not shown). IVIS^®^ *in vivo* imaging was used to image the luminescent *S. aureus* within the lungs *in vivo* (Figure 5A) and *ex vivo* (Figure 5B) 6 h after infection. Low dose propionate pre-treatment did not alter the levels of *S. aureus* present at 6 h as compared to control mice. However, the high dose propionate group displayed two- to three-fold greater levels of bacteria within the lungs (Figure 5C), suggesting that SCFA-mediated attenuation of lung inflammation prior to and during infection diminished the control of the bacterial pneumonia.

### Propionate-Producing Bacteria Are Enriched in Gut Microbiomes of Mice With Attenuated Lung Inflammatory Responses

**FIGURE 6:**
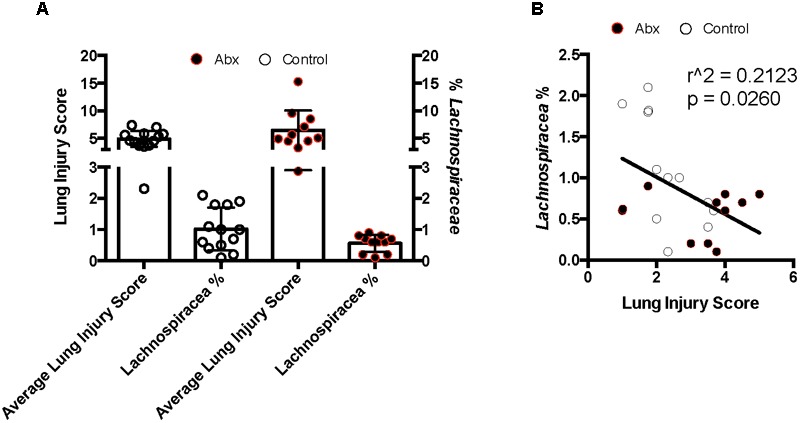
Lung inflammation after ischemia reperfusion (IR) sterile injury negatively correlates with enrichment in *Lachnospiraceae* in both control and antibiotic-treated mice. **(A)** Comparison of average lung injury score by semiquantitative lung injury scoring with percentage of *Lachnospiraceae* present as determined by 16S sequencing and taxonomic OTU determination. **(B)** Correlation of lung injury score with *Lachnospiraceae* enrichment. Pearson correlation coefficient calculated; two-tailed *p*-value presented. Control mice denoted by white circles and antibiotic treated mice by black circles.

To attempt to correlate physiologic/pathologic responses to lung IR with specific composition of resident gut microbiota within a given mouse, we subjected C57BL/6 wild type mice to an 8-week course of either control water or antibiotics (Neo/PMB) and collected stool samples weekly. At the end of 8 weeks, the mice underwent lung IR injury and lung injury was assessed by histology. Surprisingly, we observed greater than expected variation in the mice from each group vis-à-vis their lung injury (Supplementary Figure S7 and Supplementary Table ST2). To understand the source of this variation as well as the effects of the antibiotic treatment on the gut microbiome, we analyzed stool samples after 7 weeks of antibiotic-treatment and prior to lung IR surgery by 16S sequencing. Antibiotic treatment resulted in significantly reduced alpha diversity (increased evenness with reduced richness and alpha diversity by the Faith phylogenetic diversity index but not by Shannon and Simpson phylogenetic diversity indices) (Supplementary Figure S8). Firmicutes dominated the microbiome of the control group, while levels of Verrucomicrobia and Bacteriodetes were enriched after the course of antibiotics (Supplementary Figure S9). In mice that had a higher lung injury score, alpha diversity was significantly reduced (increased evenness with reduced richness and alpha diversity by the Faith and Simpson phylogenetic diversity indices) (Supplementary Figure S10 and data not shown). Beta diversity was also significantly different between the control and antibiotic treatment groups as well as between those mice that had a higher vs. lower lung injury score. Differences in bacterial composition was estimated to account for ∼15–20% of the observed lung injury differences (data not shown). Additionally, differences in bacterial community composition were significantly influenced by phylogenetic relatedness, bacterial/presence absence, relative abundance and presence of more and lesser abundant taxa (data not shown). Interestingly, specific OTUs (operational taxonomic units) were enriched (by a Three Model Approach including Negative Binomial Regression, Zero-inflated Negative Binomial Regression, and Poisson Regression with a false discovery rate corrected *p*-value < 0.2) in mice with *lower lung injury scores*. Eighty percent of the significantly enriched OTUs were members of the phylum Firmicutes, and the orders Bacteriodales and Clostridiales, with the family *Lachnospiraceae* strongly represented (Supplementary Figure S10). We observed that low lung injury correlated with higher levels of *Lachnospiraceae* (Figure 6A), and this correlation was significant (r^2^ 0.2123, Figure 6B). Coincidently, Bacteriodales and Clostridiales (specifically *Lachnospiraceae*) are the primary producers of propionate within the gut microbiome (Reichardt et al., 2014; Salonen et al., 2014; Louis and Flint, 2017). We also confirmed by metabolomic SCFA analysis that stool of mice with the highest levels of *Lachnospiraceae* (9-fold higher *Lachnospiraceae* than the comparison group) contained ∼150-fold greater levels of propionate (45 mM vs. 300 μM, data not shown).

## Discussion

The main findings of these studies indicate that levels of microbiome-derived metabolites, specifically propionate, can influence lung immune and inflammatory responses *in vivo* and *in vitro*. We began by focusing on colonic lumen filtrate (CLF) from control mice or from mice that received antibiotics given that we had previously showed that antibiotic treatment caused significant alterations in *in vivo* and *ex vivo* lung inflammation to sterile injury (Prakash et al., 2015). First, we provided evidence that LPS was one key component of CLF that resulted in immune activation of cells, and that antibiotic CLF contained less LPS and caused less immune stimulation. We observed an unexpected proinflammatory effect of micromolar concentrations of propionate and butyrate on LPS responses *in vitro.* Compared to control mice, we observed a significant enrichment of CLF propionate concentration in antibiotic-treated mice relative to acetate. Speculating that high propionate:acetate ratios *in vivo* might explain observed attenuated inflammatory responses after lung IR in antibiotic-treated mice, we exposed lung cells *in vitro* to low and high propionate:acetate ratios and reproduced pro- and anti-inflammatory modulation of LPS responses, respectively. To confirm the anti-inflammatory effect of high propionate *in vivo*, we pretreated mouse lungs with high dose propionate, which resulted in worse control of a *S. aureus* pneumonia (unlike low dose propionate). Finally, we found gut microbiome enrichment of high propionate-producing *Lachnospiraceae* among mice with reduced lung inflammation phenotypes. Collectively, the experimental data presented here strongly support a gut-lung immune axis model in which gut-derived SCFAs in combination with CAMPs in circulation profoundly influence lung physiology and immunity.

Evidence in currently published studies support the concept that gut microbiota can influence non-gut disease states. Germ-free mice often have local and systemic physiologic perturbations (reviewed in Erturk-Hasdemir and Kasper, 2013). However, these differences have largely been attributed to altered development of the immune system in the absence of early establishment and maturation of the gut commensal microbiome. Seemingly in conflict with this microbiome-driven immune development concept, other studies have shown that repopulating or altering the gut microbiome in adult mice can rapidly alter their physiology and disease processes (Shreiner et al., 2015). This suggests that highly accessible and circulating factors from the gut microbiome have the capacity to change immune responses in remote host locations (Vieira et al., 2015). Candidate factors that fit this factor profile well include microbially released or dietary metabolites. In fact, segmented filamentous bacteria which can ferment non-digestible starches to produce butyrate and propionate have been observed to confer protection in a number of disease models in mice (Gaboriau-Routhiau et al., 2009 and reviewed in Meyerholz et al., 2002; Ericsson et al., 2014).

Metabolites have been reported to have strong immunomodulatory effects on the host (Arpaia et al., 2013; Chang et al., 2014). Specific metabolites used and produced by the commensal microbiome include short, medium, and long-chain fatty acids, indoles, carbohydrates, gylcolipids, bile acids, vitamins, and other co-factors (reviewed in Shapiro et al., 2014; Levy et al., 2015). These metabolites can regulate different aspects of cell function based on engaging cognate receptors as well as epigenetically. SCFAs, namely acetate, propionate, and butyrate, have known roles as sources of energy (butyrate), effectors of epigenetic changes (propionate and butyrate), and signal transduction (acetate, propionate, and butyrate). At millimolar concentrations, both butyrate and propionate can act as histone deacetylase (HDAC) inhibitors, which silence the transcription of specific inflammatory genes (Chang et al., 2014). Butyrate, in its role as an energy source for colonocytes, can also form part of the switch that converts cells from a metabolic program based on oxidative phosphorylation to one based on glycolysis (i.e., the Warburg effect) (reviewed in Burgess, 2012; Donohoe et al., 2012). At micromolar concentrations, SCFAs engage and signal through free fatty acid receptors (FFARs) and this engagement has been shown to be important not only locally but also for immune responses in niches not directly in contact with the gut microbiome, such as synovial joints (Vieira et al., 2015) and distal lung airways (Trompette et al., 2014). Other roles, yet to be discovered, are also likely.

We propose that pulmonary immune responses may be calibrated by the levels of commensal-derived SCFAs and CAMPs that transit through the lung and result in immune priming or dampening. In support of this concept, an *in vitro* study has suggested a similar priming phenotype to low-dose SCFA that we report here (Mirmonsef et al., 2012). Other examples of low-dose SCFA effects on immune responses exist, including one in which low levels of circulating acetate specifically promote intestinal IgA responses to microbiota through FFAR2 (GPR43) (Wu et al., 2017). We further propose that low propionate:acetate levels in the lungs create immune priming to support proinflammatory responses. This priming may thus contribute to the generation of healthy baseline immune tone in the homeostatic lung. Conversely, switching to high propionate:acetate levels through gut dysbiosis may reprogram the lung and invoke pathologic or abnormal responses to sterile and infectious challenges.

Studies in mice describe how the gut microbiome is protective against pneumococcal and *S. aureus* pneumonia (Clarke et al., 2010; Gauguet et al., 2015; Brown et al., 2017). Therefore, direct and indirect mechanisms by which the gut commensal microbiome communicates with the lung are of great interest. Some key questions that still need to be answered include the identity of the free-fatty acid receptors (FFARs) that are important for these immune effects of SCFAs and whether or not those FFARs are druggable. Our data that propionate-producing *Lachnospiraceae* are significantly associated with a low lung inflammation phenotype *in vivo* confirm our *in vitro* cell culture data. Since acetate is widely produced by most if not all commensal bacteria and its levels are largely stable in circulation (Reichardt et al., 2014; Louis and Flint, 2017), alterations of propionate production, say by antibiotic exposure, may therefore significantly alter lung immune responses. We have also shown that limiting lung inflammation downstream of lung sterile reperfusion injury can result in a disseminated experimental pneumonia in mice (Tian et al., 2017). Therefore, caution must be exercised when manipulating gut microbiome lest dampening or augmenting lung inflammatory responses cause unintended consequences.

This study has its limitations. SCFAs are just one of many metabolite classes that may have physiologic and pathologic effects, and these include long-chain fatty acids, bile acids, succinate, lactate, and aromatic amino acids (Krishnan et al., 2015) – none of which were studied here. We used the SCFA composition of the stool to estimate SCFA levels in the lung and performed experiments directly administering propionate to the mouse lung. However, direct measurement of SCFA levels in mouse tissue and plasma, while technically challenging, would be more definitive. Our *in vitro* approach to study the effects of SCFAs on lung immune cell and endothelial cell responses will need *in vivo* correlation perhaps through the use of lung-specific FFAR2 and FFAR3 conditional knockout mice. We used human cells early in our studies to confirm that the effects we observed were not limited to mouse cell lines and could be translatable, but further correlation with human data is clearly required. The pulmonary microbiome contributes to lung immunity in disease states such as COPD and though unlikely may also do so in healthy lungs (Dickson et al., 2016). Finally, our work does not exclude the possibility of other members of the microbiome (besides *Lachnospiraceae*) playing important roles as well through their metabolites or released factors that could also modulate lung immunity.

We conclude that specific SCFA metabolites, namely propionate and possibly acetate, are important contributors to the gut-lung immune axis of communication that may augment and suppress lung immune responses. Specifically, high propionate:acetate levels in the lung may be beneficial in situations where lung inflammation suppression is the goal, such as following lung transplant when ischemia reperfusion injury is a threat to graft survival. On the other hand, high propionate:acetate levels that may result from poor antibiotic stewardship and resulting gut microbiome dysbiosis could adversely affect the course of bacterial pneumonia. Propionate may be an unusual non-native compound (believed to be only derivable from the diet or gut microbiome) that has perhaps been adapted for specialized functions making it a fascinating topic for further investigation. Overall, we believe that SCFA levels achieved in the pulmonary parenchyma may be critical for “healthy” or “normal” primed immune responses to lung injury and as such determine the establishment of an overall homeostatic resting lung immune tone. The identification of simple but powerful metabolites and the microbiota from which they originate as important controllers of the gut-lung immune axis may help explain the vast pathophysiologic diversity of human lung injury responses as well as the ever expanding contribution to human heath of diet, lifestyle, environment, immune history, antibiotic and medication use, and hospital-setting exposure. By expanding this knowledge base and understanding, we hope to pave the way toward devising strategies to positively modulate lung immune responses within diverse clinical scenarios.

## Ethics Statement

All mouse studies were approved from an ethical and methodological standpoint by the Institutional Animal Care and Use Committee (IACUC) at the University of California, San Francisco and followed the ARRIVE guidelines.

## Author Contributions

XT performed all *in vitro* and some *in vivo* experiments, analyzed data, and edited manuscript. JH assisted with experimental design, analyzed data, and edited manuscript. AH, HC, and KF engineered and developed the bacterial strains used in the study. AP designed all the experiments, performed mouse surgeries, analyzed data, and wrote and edited manuscript.

## Conflict of Interest Statement

The authors declare that the research was conducted in the absence of any commercial or financial relationships that could be construed as a potential conflict of interest.

## Footnotes

1 https://metabolomic.research.chop.edu/
