# Corrigendum: Elevated Gut Microbiome-Derived Propionate Levels Are Associated With Reduced Sterile Lung Inflammation and Bacterial Immunity in Mice

**DOI:** 10.3389/fmicb.2019.00518

**Published:** 2019-03-26

**Authors:** Xiaoli Tian, Judith Hellman, Alexander R. Horswill, Heidi A. Crosby, Kevin P. Francis, Arun Prakash

**Affiliations:** ^1^Department of Anesthesia and Perioperative Care, University of California, San Francisco, San Francisco, CA, United States; ^2^Department of Immunology and Microbiology, Anschutz Medical Campus, University of Colorado, Aurora, CO, United States; ^3^Preclinical Imaging, PerkinElmer, Hopkinton, MA, United States; ^4^San Francisco General Hospital, University of California, San Francisco, San Francisco, CA, United States

**Keywords:** lung injury, short-chain fatty acids, SCFA, acetate, propionate, IR, inflammation

“Alexander R. Horswill,” “Heidi A. Crosby,” and “Kevin P. Francis” were not included as authors in the published article. The corrected **Author Contributions statement** and **Acknowledgements** appears below.

**Author Contributions**

“XT performed all *in vitro* and some *in vivo* experiments, analyzed data, and edited manuscript. JH assisted with experimental design, analyzed data, and edited manuscript. AH, HC, and KF engineered and developed the bacterial strains used in the study. AP designed all the experiments, performed mouse surgeries, analyzed data, and wrote and edited manuscript.”

**Acknowledgements**

“We would like to acknowledge the following individuals for assistance with providing reagents, mice, advice, helpful discussions, and critical reading and editing of the manuscript: Douglas Fadrosh (UCSF), Susan Lynch (UCSF), Michael Matthay (UCSF), Mervyn Maze (UCSF), Clifford Lowell (UCSF), Kevin Wilhelmsen (MarinBiologics), Milo Vassallo (Methodist Hospital, Brooklyn, NY), Gautam Prakash (Arlington, VA). The authors would also like to acknowledge the role of THFC (COYS) on these studies.”

The authors apologize for this error and state that this does not change the scientific conclusions of the article in any way. The original article has been updated.

