# Supplementary material for: Elevated Gut Microbiome-Derived Propionate Levels Are Associated With Reduced Sterile Lung Inflammation and Bacterial Immunity in Mice
Source: Front Microbiol. 2019 Feb 14;10:159. doi: 10.3389/fmicb.2019.00159 (PMC6413706; doi:10.3389/fmicb.2019.00159)
Supplement: Supplementary file 1 [file Data_Sheet_1.pdf]

Tian et al.
Supplemental Figures

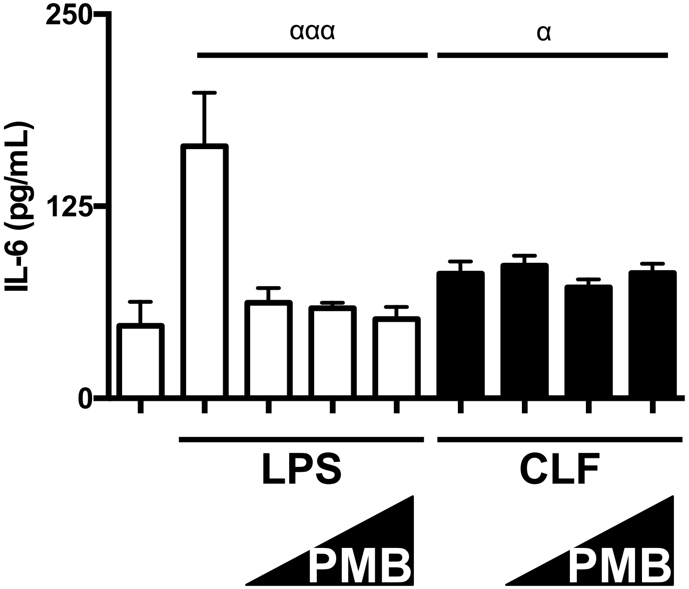

Figure S1. Human Umbilical Vein Endothelial Cells (HUVEC) respond to CLF but inflammatory response is not blocked by polymyxin B (PMB). HUVEC were treated with LPS (10μg/mL) or CLF (10μg/mL, from wild type C57BL/6 mice) and increasing amounts of polymyxin B were also co-administered (1ng/mL, 10ng/mL, and 100ng/mL). IL-6 levels measured by ELISA after overnight treatment.

Supplemental Figure S1

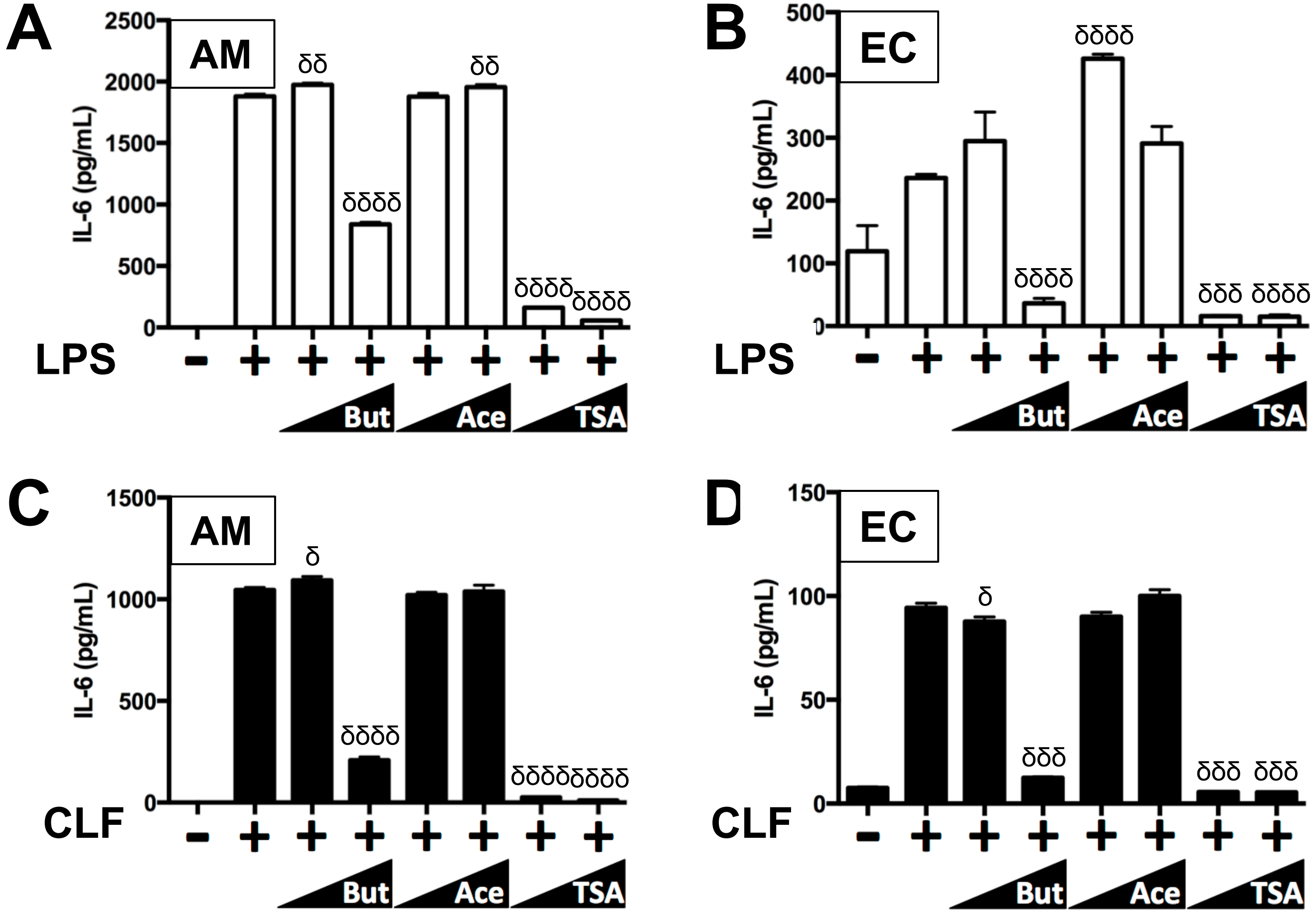

Figure S2. CLF mirrors LPS in its effects on mouse alveolar macrophage (AM) and HUVEC (EC) vis-à-vis HDAC inhibition by high concentration butyrate and trichostatin A (TSA), but not acetate. IL-6 levels from AM (A, C) and HUVEC (B, D) after being stimulated with LPS (A, B; 100ng/mL) and CLF (C, D; 50mcg/mL) from WT C57BL/6 mice. Cells were also co-incubated with low and high-dose butyrate (But; 0.2mM and 2mM), acetate (Ace; 0.1mM and 1mM), and TSA (10nM and 50nM). For comparisons against an untreated or control condition (e.g. in (A) and (B) LPS treatment only and in (C) and (D) CLF treatment only), p values are represented as follows: δ< 0.05; δδ< 0.01; δδδ< 0.001; δδδδ< 0.0001. All experiments were performed at least twice and representative data are shown.

Supplemental Figure S2

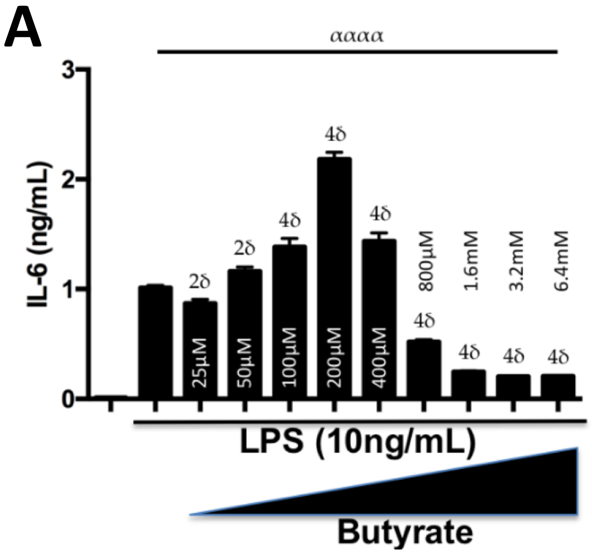

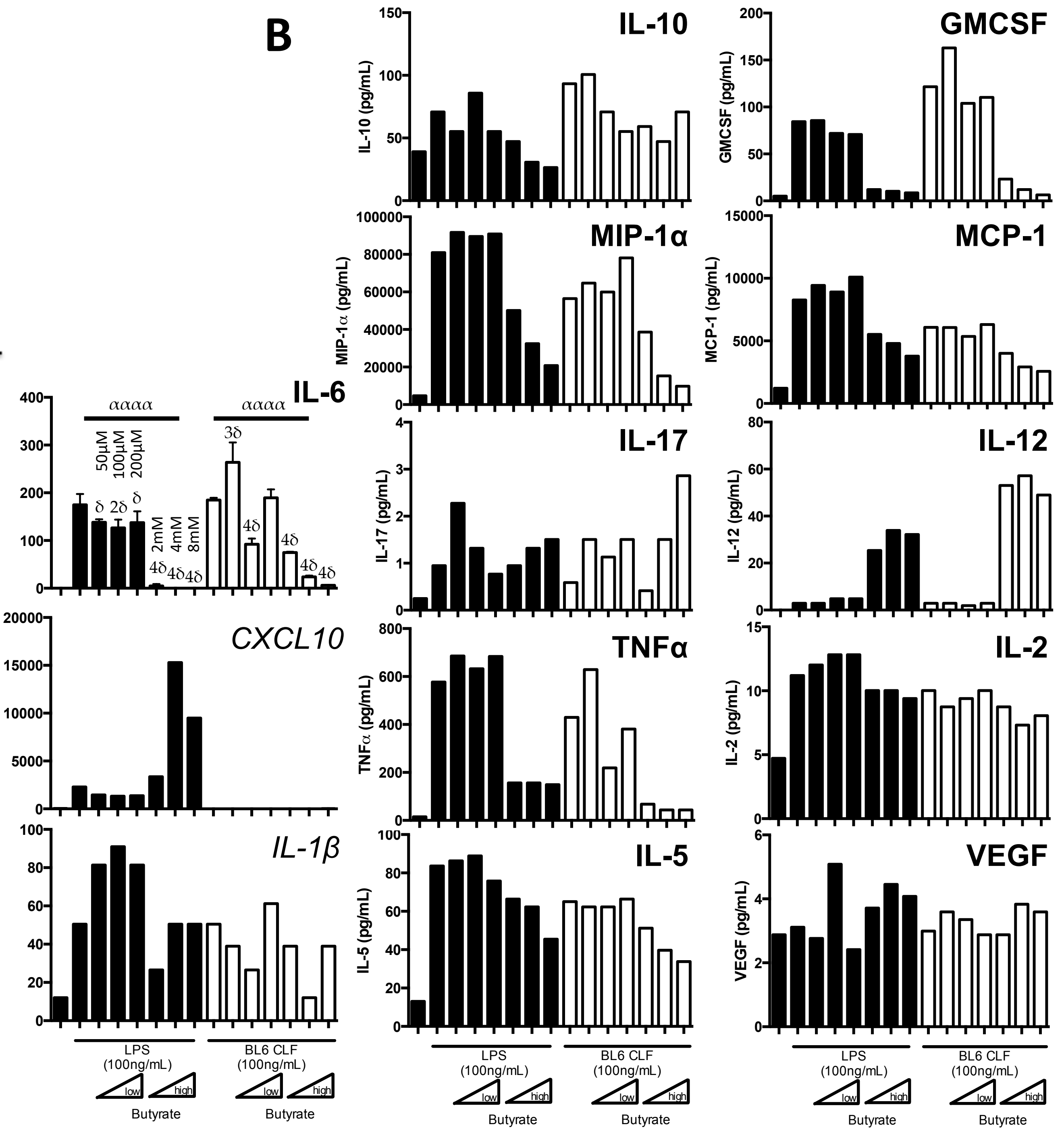

Figure S3. Analyzing the low and high dose effects of butyrate on a battery of immune gene products produced when AMs are challenged with LPS or CLF.

(A) IL-6 levels after LPS challenge and wide range of butyrate doses.

(B) Immune genes regulated similarly by butyrate after LPS and CLF challenge (bold). Immune genes regulated differently by butyrate after LPS and CLF challenge (italics).

Supplemental Figure S3

mM	C57 BL/6 WT CLF	C3H WT CLF	C3H WT CLF (+Abx)
Formic Acid	21.492	94.995	92.8875
Acetic Acid	19.2155	35.873	6.49
Propionic Acid	1.349	1.9695	32.209
Butyric Acid	13.3665	19.4235	17.745
Isobutyric Acid	ND	ND	ND
Isovaleric Acid	3.4525	7.6835	4.131
Valeric Acid	0.848	2.995	2.385
Caproic Acid	0.131	0.8845	0.6565
Heptanoic Acid	ND	2.506	11.4635

ND = not detected

Concentration (µM)	His	Ser	Arg	Gly	Asp	Glu	Thr	Ala	Phe
C57 BL/6 WT CLF	25.933	95.036	108.17	140.903	96.084	171.919	77.318	11.189	28.103
C3H WT CLF	26.616	91.145	108.735	123.249	132.296	203.59	84.297	13.909	35.539
C3H WT CLF (+Abx)	26.604	111.231	135.903	149.16	109.54	160.64	92.666	10.818	29.481

	Pro	Cys	Lys	Tyr	Met	Val	Ile	Leu
C57 BL/6 WT CLF	78.079	1.417	70.269	52.123	9.227	127.019	68.606	146.054
C3H WT CLF	87.609	1.336	94.385	61.178	18.494	136.524	85.156	177.22
C3H WT CLF (+Abx)	128.464	1.83	73.338	49.419	12.588	133.406	77.118	155.327

Table ST1. Measured levels of SCFAs (including medium and branch chain fatty acids) and amino acids from Colonic lumen filtrate (CLF) C57BL/6 wildtype, C3H/HeOuJ wildtype mice treated with control water and C3H/HeOuJ wildtype mice treated with antibiotic (+Abx) water.

Supplemental Table ST1

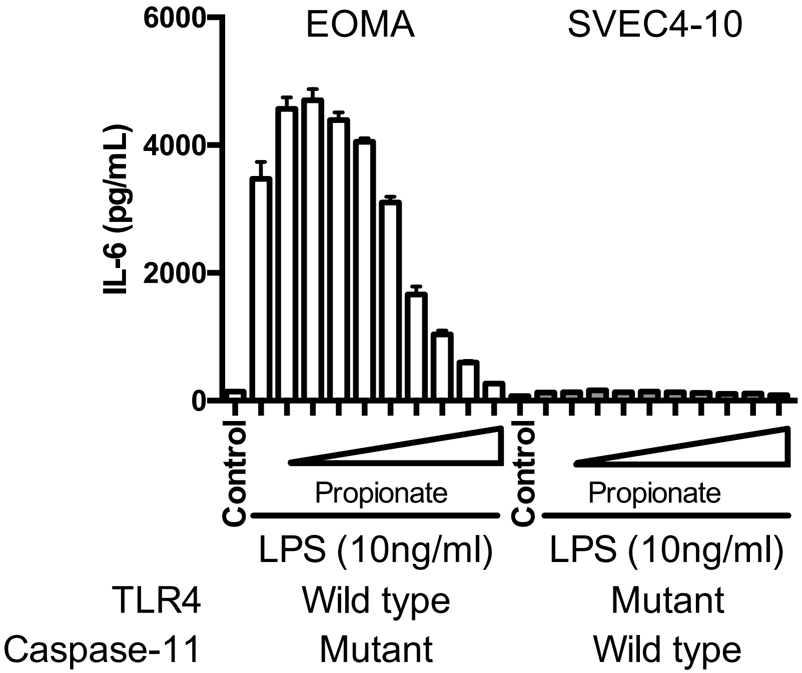

Figure S4. TLR4 (and not caspase-11) sensing of LPS is required for propionate effects. EOMA (left, 129 background, TLR4 wild type, Caspase-11 mutant), and SVEC4-10 (right, C3H/HeJ background, TLR4 mutant, Caspase-11 wild type) endothelial cell lines were treated with a wide dose range of propionate (25μM-6.4mM) either in the presence or absence of LPS (10ng/mL) and overnight production of IL-6 by ELISA was measured.

Supplemental Figure S4

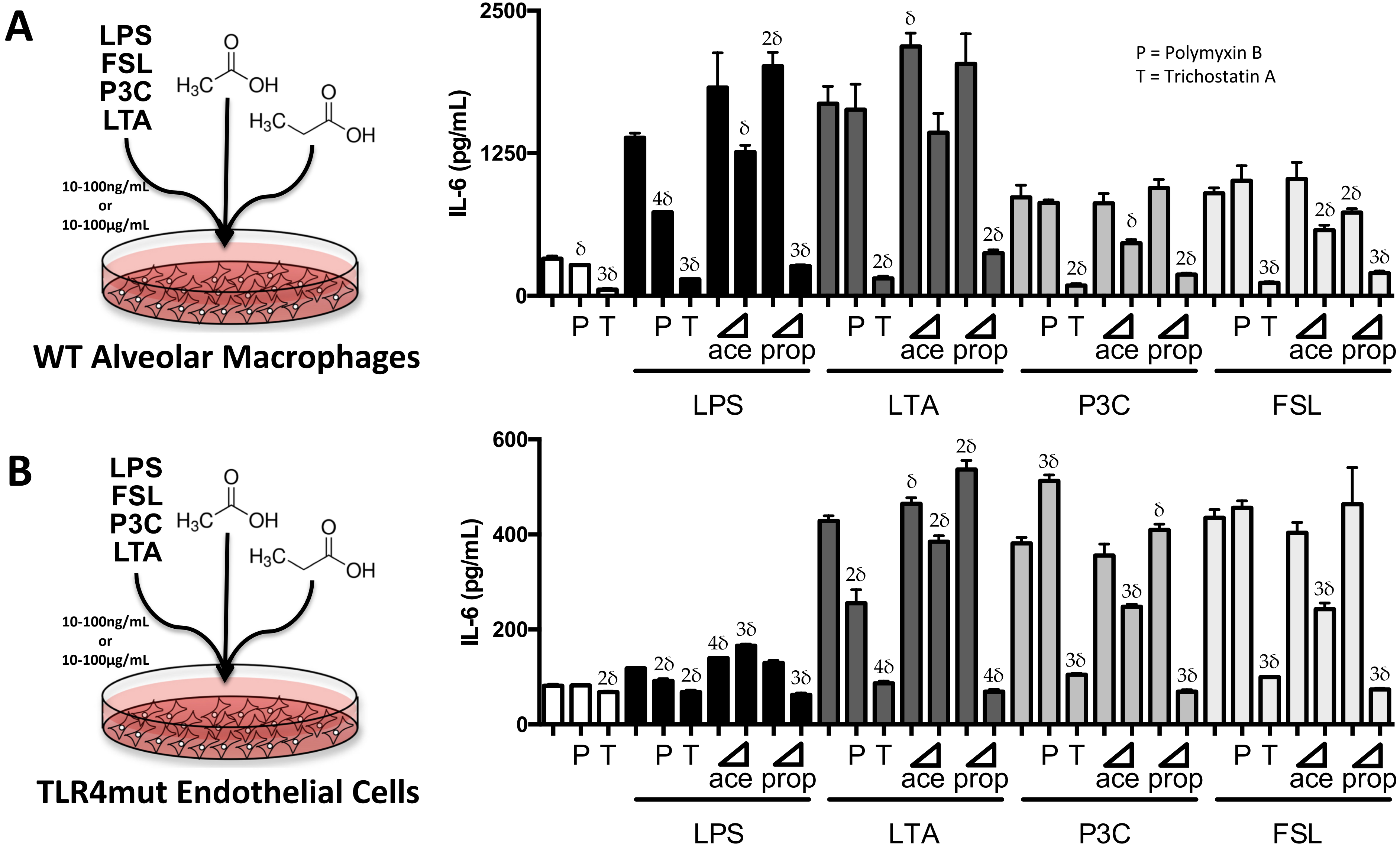

Figure S5. The inflammatory response to TLR2 ligands can also be enhanced or sustained by acetate and low-dose propionate and inhibited by high-dose propionate. (A) MH-S WT AMs or (B) SVEC4-10 TLR4mut ECs or challenged with either LPS or LTA, P3C, FSL (all TLR2 ligands) in the presence of low and high acetate and propionate, polymyxin B, or trichostatin A. After overnight incubation, IL-6 levels were measured. PolymyxinB (P) used as a LPS inhibitor and Trichostatin A (T) as an HDAC inhibitor.

Supplemental Figure S5

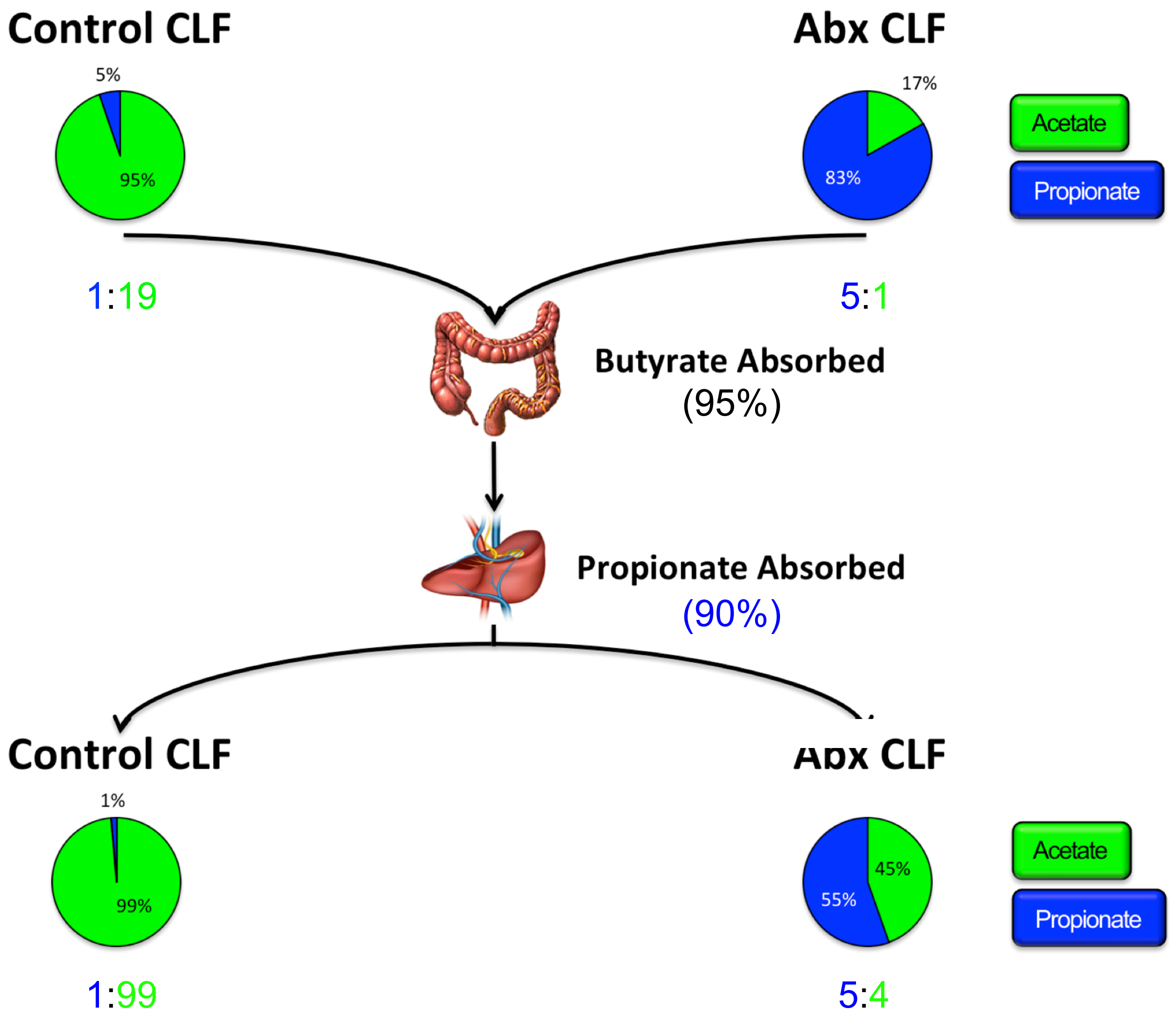

Figure S6. Differences in propionate:acetate ratios between control and antibiotic-exposed CLF are present in peripheral blood even after accounting for absorption by gut and liver. Ratios of the SCFAs are represented with acetate denoted in green and propionate in blue. Based on the estimates for intestinal and liver absorption reported by Boets et al. (2015), namely 90% propionate and 95% butyrate absorption, the estimated changes in ratios of SCFAs between the CLF and peripheral circulation (and the pulmonary circulation) are shown.

Supplemental Figure S6

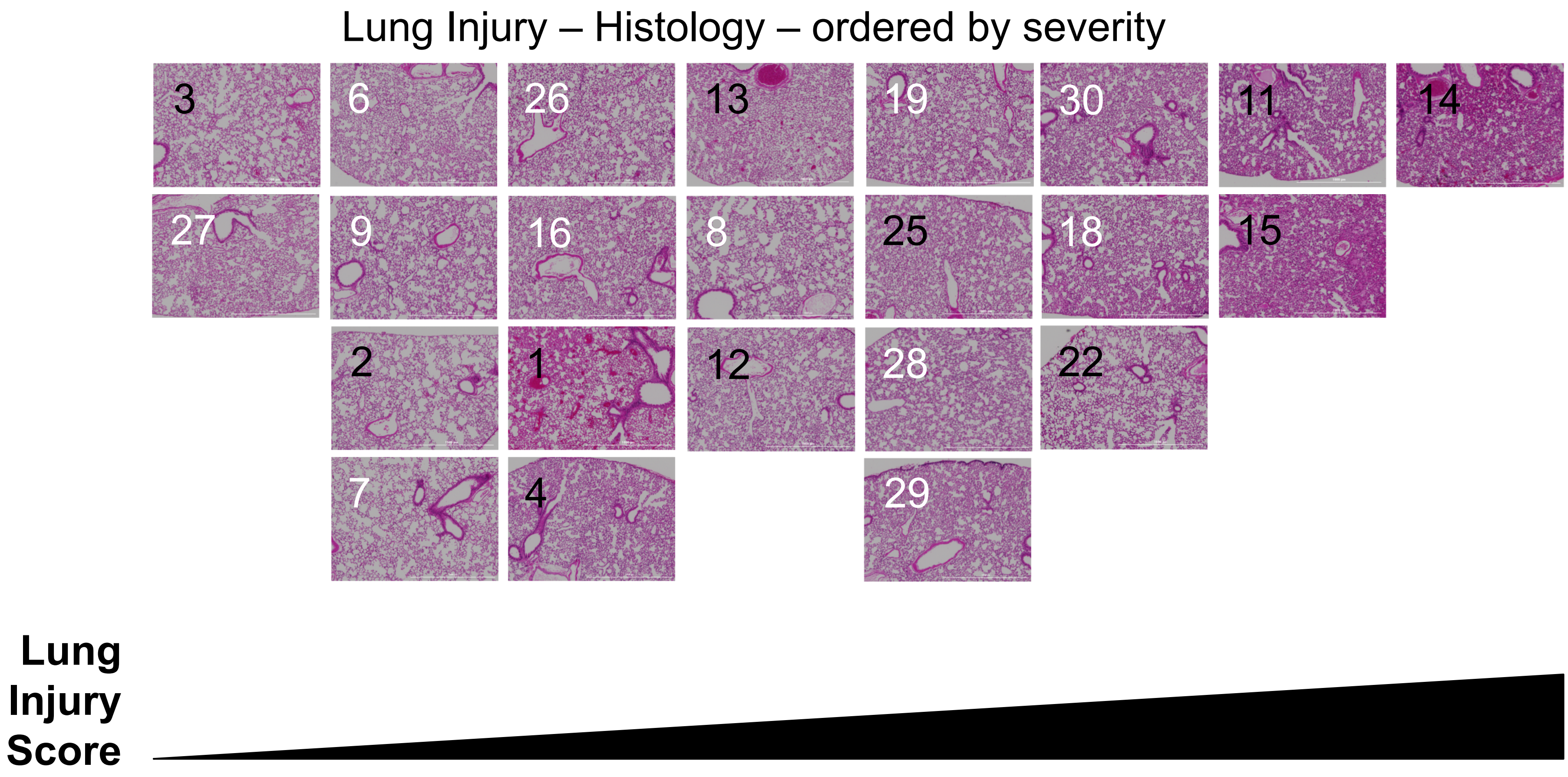

Figure S7. Left lung injury assessment by histology after 3h of reperfusion following 1h of left lung ischemia; histological images have been ordered from left to right by increasing lung injury score (LIS), as determined by average %area (see Supplemental Figure ST2). Lungs from mice that received control water are numbered in white text, and mice that received antibiotic water in black text.

Lung Injury Scoring

Mouse ID	Average Lung Injury Score	Average %area	Average count
401 (29)	3.666666667	5.592	7328
402 (28)	2.333333333	5.737	8034.5
403 (27)	2.666666667	2.307	4975
404 (30)	3.5	6.983	7963
405 (26)	1.75	4.587	5622.5
406 (18)	3.5	7.3435	8265
408 (19)	2.333333333	5.8345	6571.5
410 (16)	2	4.317	5663
411 (6)	2	3.6555	5620.5
412 (9)	1.75	3.5145	5410
413 (7)	1	3.7	5002
415 (8)	1.75	5.2685	5695.5
418 (22)	3.5	7.068	6170.5
420 (25)	3.75	5.6575	5535.5
421 (13)	4	4.9145	6056.5
422 (11)	4	9.5165	6735
423 (12)	1.75	5.071	5880
424 (15)	4.5	8.511	6714.5
425 (14)	5	15.272	8388
427 (4)	3	4.504	6373
428 (2)	1	3.2695	4412
429 (3)	1	2.867	4082
430 (1)	3.75	4.489	4670.5
Average	2.760869565	5.651282609	6137.73913
Cutoff	2.67	5.592	6056.5

Table ST2. Lung Injury Scores (LIS) for mice after lung IR. This table supports the data shown in Figure S8. Average Lung injury score refers to the semi-quantitative measurement as previously described (12); average %area and average count are measured using ImageJ as described in the methods section. Red cells = high injury score.

Supplemental Table ST2

Alpha diversity

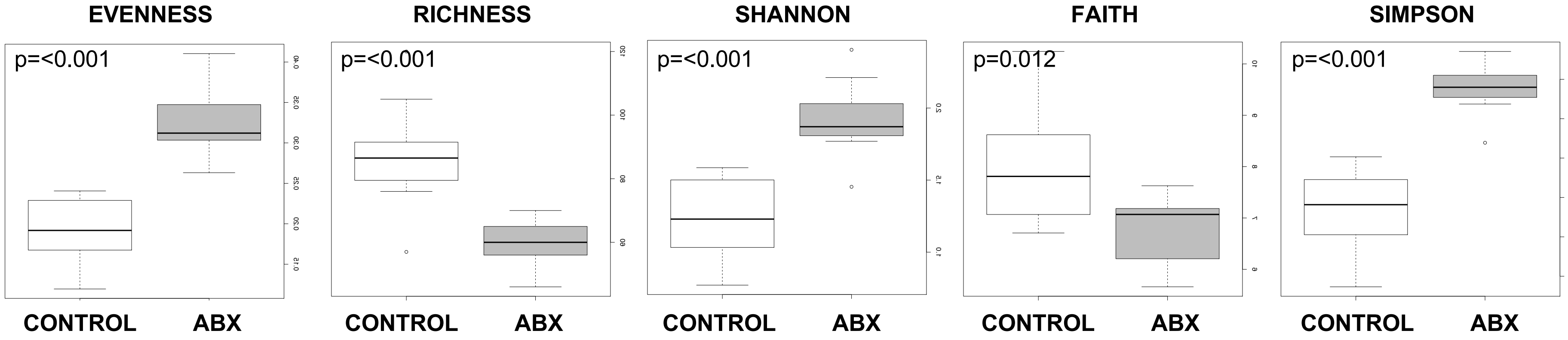

Figure S8. 16S microbiome profiling comparing alpha diversity of the control group vs. antibiotic group. Alpha diversity is represented in terms of Evenness, Richness, as well as Shannon, Faith and Simpson Phylogenetic Diversity Indices. None of the differences in alpha diversity are significant at week 1 while all are significant at week 7 by Kruskal-Wallis and ANOVA with p values all <0.001 except for Faith's Phylogenetic Diversity Index (0.012 & 0.004). K-W p values denoted.

Supplemental Figure S8

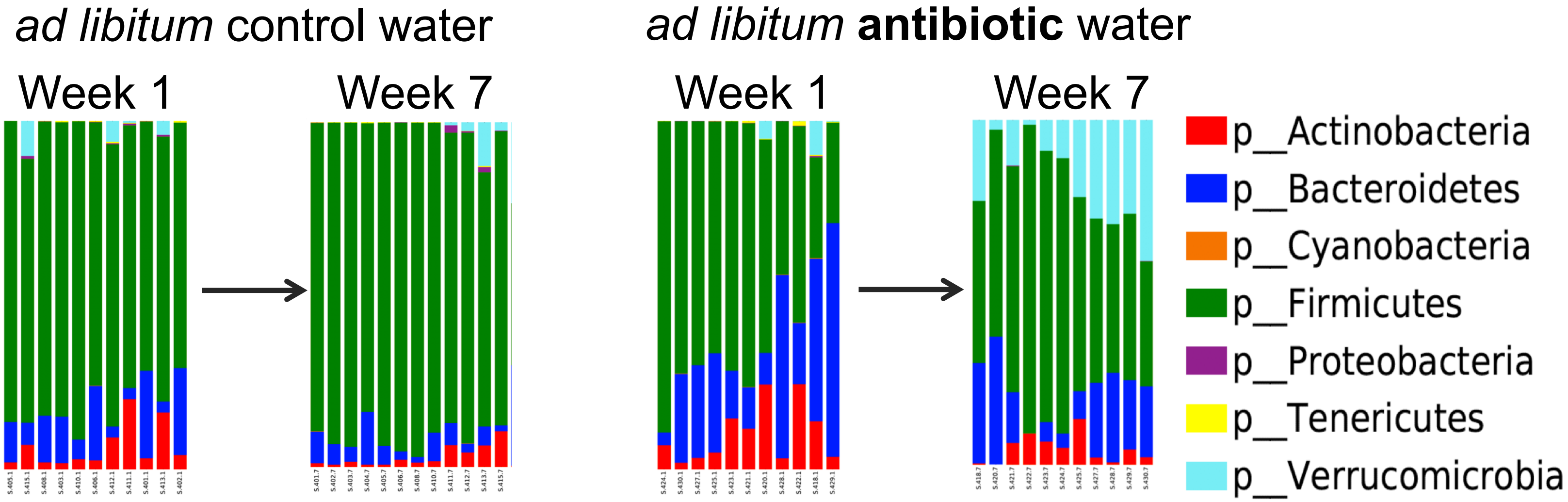

Figure S9. Two cohorts of wild type C3H mice were either given control drinking or antibiotic water containing Neomycin/Polymyxin B for 8 weeks. Stool pellets were collected weekly and at week 8, mice received left lung IR surgery. Stool from week 1 and 7 were processed for 16S microbiome profiling and phylum-level enrichment is presented. 16S sequencing and profiling for one sample from week 1 (control water) failed.

Supplemental Figure S9

Week 7 Alpha diversity based on Average Lung Injury Scoring (LIS)

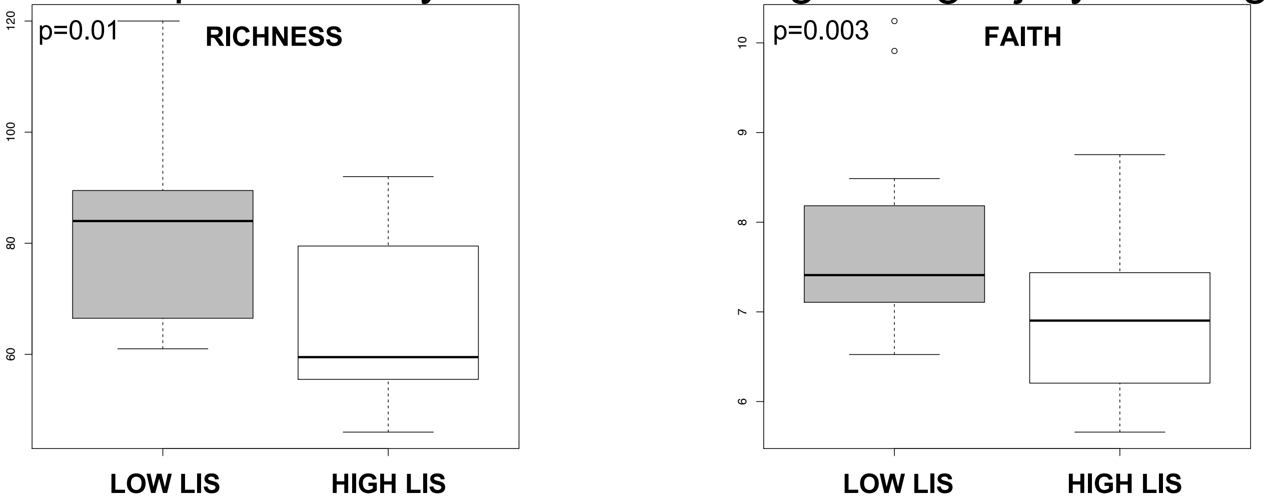

	OTUname	pois.pval	nb.pval	zinb.pval	best.mod	qval.best	mean_diff	zerotrt1	zerotrt2	nonzerotrt1	nonzerotrt2	totaltrt1	totaltrt2	taxonomy
**Enriched in HIGH lung Injury Group**														
	OTUname	pois.pval	nb.pval	zinb.pval	best.mod	qval.best	mean_diff	zerotrt1	zerotrt2	nonzerotrt1	nonzerotrt2	totaltrt1	totaltrt2	taxonomy
94	OTU_41	7.86E-18	0.025	0.06686	NegBin	0.16317	-14.5985	6	9	5	3	9	185	k__Bacteria; p__Firmicutes; c__Clostridia; o__Clostridiales; f__Ruminococcaceae; g__; s__
54	OTU_244	4.13E-05	0.252	0.01252	ZI-NegBin	0.12457	-2.84848	8	9	3	3	9	44	k__Bacteria; p__Firmicutes; c__Bacilli; o__Turicibacterales; f__Turicibacteraceae; g__Turicibacter; s__
**Ennriched in LOW Lung Injury Group**														
	OTUname	pois.pval	nb.pval	zinb.pval	best.mod	qval.best	mean_diff	zerotrt1	zerotrt2	nonzerotrt1	nonzerotrt2	totaltrt1	totaltrt2	taxonomy
2	OTU_10	0	NA	#######	ZI-NegBin	0.00373	372.3864	3	6	8	6	4539	483	k__Bacteria; p__Bacteroidetes; c__Bacteroidia; o__Bacteroidales; f__Rikenellaceae; g__; s__
122	OTU_7	1.06E-146	0.054	0.02824	ZI-NegBin	0.1649	138.3712	0	3	11	9	2480	1045	k__Bacteria; p__Bacteroidetes; c__Bacteroidia; o__Bacteroidales; f__S24-7; g__; s__
78	OTU_31	6.48E-65	NA	0.00096	ZI-NegBin	0.02985	62.56061	8	11	3	1	712	26	k__Bacteria; p__Proteobacteria; c__Epsilonproteobacteria; o__Campylobacterales; f__Helicobacteraceae; g__Helicobacter; s__
31	OTU_15	8.29E-73	0.015	0.0135	NegBin	0.13648	50.38636	2	3	9	9	678	135	k__Bacteria; p__Firmicutes; c__Clostridia; o__Clostridiales; f__; g__; s__
109	OTU_57	2.26E-10	2E-04	0.00013	NegBin	0.0118	15.01515	5	11	6	1	167	2	k__Bacteria; p__Firmicutes; c__Clostridia; o__Clostridiales; f__Lachnospiraceae; g__[Ruminococcus]; s__gnavus
60	OTU_27	3.43E-19	0.116	0.00763	ZI-NegBin	0.11217	13.31818	5	6	6	6	196	54	k__Bacteria; p__Actinobacteria; c__Coriobacteriia; o__Coriobacteriales; f__Coriobacteriaceae; g__Adlercreutzia; s__
104	OTU_52	3.21E-15	0.005	0.00482	NegBin	0.08802	9.651515	3	7	8	5	119	14	k__Bacteria; p__Firmicutes; c__Erysipelotrichi; o__Erysipelotrichales; f__Erysipelotrichaceae; g__; s__
99	OTU_47	8.25E-08	NA	0.0128	ZI-NegBin	0.12457	7.227273	7	9	4	3	151	78	k__Bacteria; p__Firmicutes; c__Bacilli; o__Lactobacillales; f__Streptococcaceae; g__Streptococcus; s__
128	OTU_77	1.02E-09	0.019	#######	ZI-NegBin	5.30E-05	5.962121	7	8	4	4	72	7	k__Bacteria; p__Firmicutes; c__Clostridia; o__Clostridiales; f__Lachnospiraceae; g__; s__
83	OTU_33	4.91E-07	0.017	0.05952	NegBin	0.14812	5.871212	0	3	11	9	115	55	k__Bacteria; p__Firmicutes; c__Clostridia; o__Clostridiales; f__Ruminococcaceae; g__Oscillospira; s__
88	OTU_34	6.68E-07	0.034	0.03372	NegBin	0.18929	5.55303	0	3	11	9	106	49	k__Bacteria; p__Firmicutes; c__Clostridia; o__Clostridiales; f__; g__; s__
95	OTU_42	6.67E-06	0.028	0.0281	NegBin	0.1649	3.484848	3	5	8	7	53	16	k__Bacteria; p__Firmicutes; c__Clostridia; o__Clostridiales; f__Lachnospiraceae; g__; s__
138	OTU_87	2.09E-05	0.002	0.00186	NegBin	0.05658	3.469697	5	10	6	2	40	2	k__Bacteria; p__Firmicutes; c__Clostridia; o__Clostridiales; f__; g__; s__
102	OTU_50	2.42E-06	0.021	0.0205	NegBin	0.15427	3.431818	4	8	7	4	46	9	k__Bacteria; p__Firmicutes; c__Clostridia; o__Clostridiales; f__; g__; s__
135	OTU_84	7.94E-06	0.023	0.00207	NegBin	0.15813	3.045455	6	8	5	4	39	6	k__Bacteria; p__Firmicutes; c__Clostridia; o__Clostridiales; f__Lachnospiraceae; g__Dorea; s__
117	OTU_64	8.51E-05	0.037	0.03653	NegBin	0.19759	2.742424	2	7	9	5	43	14	k__Bacteria; p__Firmicutes; c__Clostridia; o__Clostridiales; f__; g__; s__
96	OTU_43	0.00013	0.005	0.00365	NegBin	0.08802	2.560606	5	11	6	1	30	2	k__Bacteria; p__Firmicutes; c__Clostridia; o__Clostridiales; f__; g__; s__
115	OTU_62	8.31E-05	0.048	0.00095	NegBin	0.24006	2.416667	7	6	4	6	33	7	k__Bacteria; p__Firmicutes; c__Erysipelotrichi; o__Erysipelotrichales; f__Erysipelotrichaceae; g__; s__
121	OTU_69	0.000681	0.012	0.01232	NegBin	0.12457	1.977273	2	7	9	5	30	9	k__Bacteria; p__Firmicutes; c__Clostridia; o__Clostridiales; f__Ruminococcaceae; g__; s__
57	OTU_250	0.001867	0.106	0.00975	ZI-NegBin	0.12457	1.916667	5	6	6	6	33	13	k__Bacteria; p__Verrucomicrobia; c__Verrucomicrobiae; o__Verrucomicrobiales; f__Verrucomicrobiaceae; g__Akkermansia; s__muciniphila
106	OTU_54	0.020286	0.023	NA	Poisson	0.15427	1.893939	0	0	11	12	52	34	k__Bacteria; p__Firmicutes; c__Bacilli; o__Lactobacillales; f__Streptococcaceae; g__Streptococcus; s__
118	OTU_65	0.02059	NA	0.00102	ZI-NegBin	0.02985	1.636364	6	5	5	7	40	24	k__Bacteria; p__Firmicutes; c__Clostridia; o__Clostridiales; f__Ruminococcaceae; g__Ruminococcus; s__
20	OTU_123	0.00454	0.008	0.00768	NegBin	0.11217	1.462121	4	11	7	1	17	1	k__Bacteria; p__Firmicutes; c__Clostridia; o__Clostridiales; f__Ruminococcaceae; g__Oscillospira; s__
38	OTU_184	0.002589	0.02	0.01995	NegBin	0.15427	1.386364	4	10	7	2	18	3	k__Bacteria; p__Firmicutes; c__Clostridia; o__Clostridiales; f__; g__; s__
129	OTU_78	0.010604	0.013	0.01278	Poisson	0.12457	1.098485	4	11	7	1	13	1	k__Bacteria; p__Firmicutes; c__Clostridia; o__Clostridiales; f__Ruminococcaceae; g__; s__
132	OTU_80	0.025704	0.028	0.0281	Poisson	0.16317	0.954545	2	6	9	6	16	6	k__Bacteria; p__Firmicutes; c__Clostridia; o__Clostridiales; f__Ruminococcaceae; g__; s__
24	OTU_131	0.041097	0.062	0.05649	Poisson	0.21429	0.643939	7	11	4	1	8	1	k__Bacteria; p__Firmicutes; c__Clostridia; o__Clostridiales; f__Lachnospiraceae; g__; s__

Figure S10. Alpha diversity and Taxonomic enrichment in low vs. high lung injury groups as defined by average lung injury score (LIS) (as described in table ST2). All Clostridiales are highlighted in yellow and all Bacteriodales are highlighted in orange. Differences in alpha diversity are significant ($p<0.05$) between low and high LIS at week 7 for richness, evenness, and all phylogentic diversity indices by Kruskal-Wallis (K-W) and ANOVA (except K-W p=0.065 for Shannon diversity index). K-W p values denoted.

Supplemental Figure S10
